# Universal ingredients to parenting teens: parental warmth and autonomy support promote adolescent well-being in most families

**DOI:** 10.1038/s41598-022-21071-0

**Published:** 2022-10-07

**Authors:** Anne Bülow, Andreas B. Neubauer, Bart Soenens, Savannah Boele, Jaap J. A. Denissen, Loes Keijsers

**Affiliations:** 1 Department of Psychology, Education and Child Studies, Erasmus University Rotterdam, P.O. Box 1738, 3000 DR Rotterdam, The Netherlands; 2 DIPF|Leibniz Institute for Research and Information in Education, Frankfurt, Germany; 3 Department of Developmental, Personality and Social Psychology, Ghent University, Ghent, Belgium; 4 Department of Developmental Psychology, Utrecht University, Utrecht, The Netherlands

**Keywords:** Psychology, Risk factors

## Abstract

Even though each adolescent is unique, some ingredients for development may still be universal. According to Self-Determination Theory, every adolescent’s well-being should benefit when parents provide warmth and autonomy. To rigorously test this idea that each family has similar mechanisms, we followed 159 Dutch parent-adolescent dyads (parent: *M*_*age*_ = 45.34, 79% mothers; adolescent: *M*_*age*_ = 13.31, 62% female) for more than three months, and collected 100 consecutive daily reports of parental warmth, autonomy support, positive and negative affect. Positive effects of parental warmth and autonomy support upon well-being were found in 91–98% of the families. Preregistered analysis of 14,546 daily reports confirmed that effects of parenting differed in strength (i.e., some adolescents benefited more than others), but were universal in their direction (i.e., in fewer than 1% of families effects were in an unexpected direction). Albeit stronger with child-reported parenting, similar patterns were found with parent-reports. Adolescents who benefited most from need-supportive parenting in daily life were characterized by higher overall sensitivity to environmental influences. Whereas recent work suggests that each child and each family have unique developmental mechanisms, this study suggests that need-supportive parenting promotes adolescent well-being in most families.

## Introduction

Although each child is unique, some of the contextual influences needed for children to thrive may be universal. Whereas there is consensus among developmental scholars that parenting is an important contextual influence on children’s and adolescents’ well-being^1^, it is still debated whether a universal recipe for high-quality parenting exists^2,3^. Self-Determination Theory^4^ posits that certain behaviors of parents are need-supportive and thus universally beneficial for children’s and adolescents’ well-being and development. Specifically, parental warmth (which makes adolescents feel loved and cared for) and parental autonomy support (which allows adolescents to feel they can take initiative and be authentic) should satisfy each adolescent’s basic psychological needs, and therefore contribute to *every* adolescents’ well-being, although the strength of these effects may differ between adolescents^2,5^. In other words, if indeed parental warmth and autonomy support are universal ingredients, we would expect that positive effects are found across cultures^6^, and within cultures^7^, in (almost) all families. Negative effects should be practically absent.

Whereas some studies have provided evidence for this universality claim by comparing different cultures^6^, it is yet to be tested whether each individual adolescent benefits. This study assesses, for the first time, in how many families these assumed universal benefits of need-supporting parenting apply. For this purpose, the current study employed a novel family-specific approach that relies on intensive repeated assessments (i.e., daily measures across 100 consecutive days)—which allowed us to estimate, for each participating family, their unique (i.e., idiographic) association between need-supportive parenting and adolescents’ affective well-being^8,9^ (in terms of positive and negative affect).

### A self-determination perspective on parenting

Self-Determination Theory (SDT)^4,10,11^ is a broad theory about human motivation and development with direct implications for parenting^5,12^. It assumes three basic (i.e., essential, and universal) needs for psychological growth and well-being: the needs for relatedness (feeling connected to others), autonomy (feeling a sense of volition and authenticity), and competence (feeling capable and effective). Extensive empirical research has shown that the satisfaction of these basic human needs is associated with better well-being and better psychosocial functioning across life domains (e.g., work/school, sports/hobbies, relationships) and across different developmental periods, including adolescence^4^. This fundamental role of psychological need satisfaction in fostering adolescent well-being has been demonstrated at different conceptual levels of analysis. At the between-person level, adolescents who experience more need satisfaction, compared to others, also report higher well-being than others^13^. At the within-person level, on days when an adolescent’s needs are more satisfied than usual, they generally feel better^14^. In sum, there is strong support for the importance of need satisfaction.

SDT describes three basic needs as essential nutrients for psychosocial growth, much like a plant needs soil, water, and sunshine. Parents can be seen as gardeners providing these nutrients for their children to flourish^15,16^. A large body of research^5,12,17^ suggest that parents can contribute to (or undermine) need satisfaction in (at least) two ways. *Parental warmth*, which involves interacting with a child in an affectionate and responsive way, mainly fosters relatedness need satisfaction; and *autonomy support*, which entails recognizing the child’s perspective and encouraging initiative primarily fosters autonomy need satisfaction.

### Uniform, universal, or unique parenting processes

**Figure 1:**
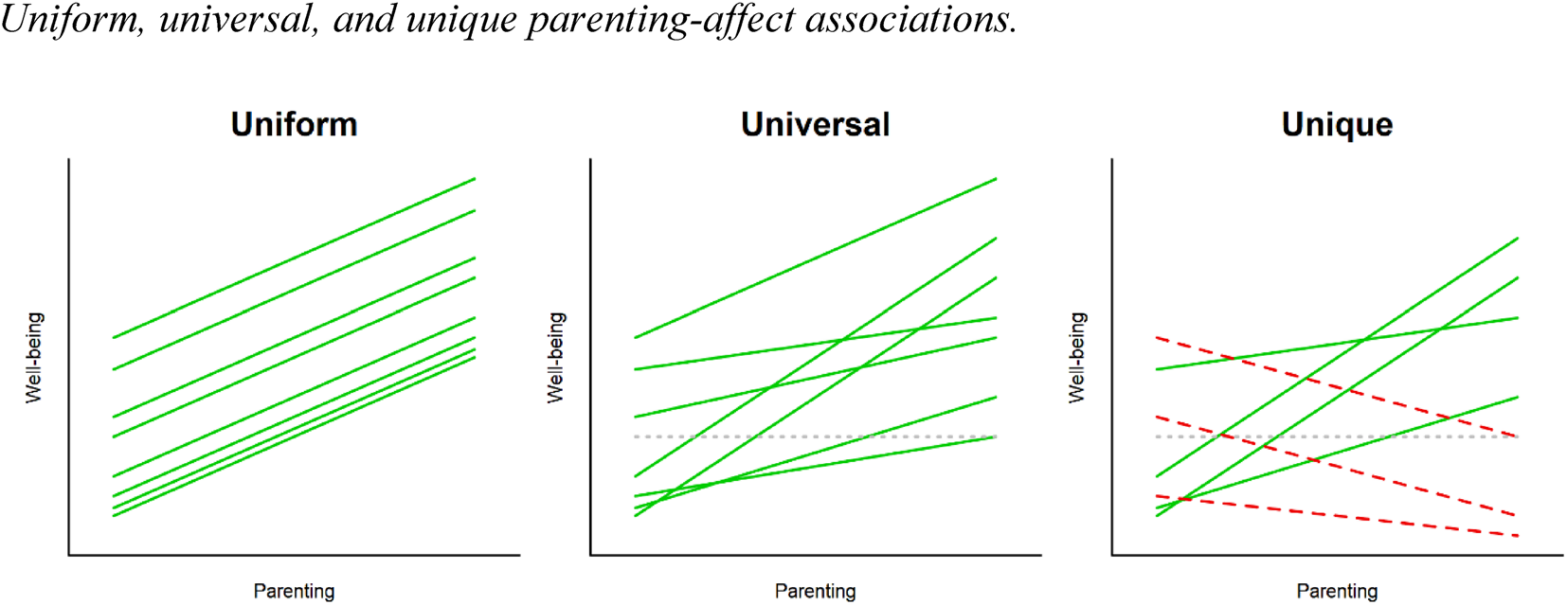
Note: Each line represents the association between parenting and well-being for one family. Green solid lines indicate a positive association, grey dotted lines indicate a null association, and red dashed lines indicate a negative association.

The question whether parental warmth and autonomy support are universally beneficial, and thus positive effects can be expected in each family, is currently fiercely discussed in the parenting literature. Currently, there are three distinct views (illustrated in Fig. 1). Firstly, based on a simplistic interpretation of SDT’s claim that the three basic needs are innate, essential, and universal for all humans^10,18^, it could be argued that all children and adolescents *uniformly* benefit (i.e., to the same extent) from parental warmth and autonomy support. Secondly, on the other side of the continuum, the idea of universality has been challenged by extreme relativistic perspectives, claiming that no parenting dimension is inherently adaptive or maladaptive^3^. Accordingly, the effects of warmth and autonomy support upon adolescent well-being would be *unique* for each adolescent. Depending on an extensive list of moderators the same parenting practice could have beneficial effects for one adolescent, and harmful effects for another^19,20^.

In between these two opposing interpretations (i.e., the simplistic uniform account and the extreme relativistic position) SDT actually holds an intermediate view referred to as *universalism without uniformity*^2,18,21^. In this perspective, parental warmth and autonomy support are still *universally* beneficial for every adolescent’s well-being, but the strength of this positive association is not uniform and thus differs between families.

How strongly adolescents are affected by need-supportive parenting may be explained by how adolescents appraise and perceive the behaviors of their parents^2^. With regard to the appraisal, some adolescents are more inclined to interpret a well-intended parental attempt to support psychological needs in a positive light, thereby actually experiencing the parental behavior as need-supportive. Other adolescents may also prefer this parenting practice over need-thwarting practice while having a somewhat less benign interpretation of the same parental behavior. For example, they might experience parents’ provision of choice as a parental attempt to promote independence when they would prefer to receive more guidance, thereby feeling somewhat left alone^22^. With regard to adolescents’ perception of parenting, a group of Environmental Sensitivity Theories^23,24^ suggest dispositional differences between children and adolescents in their overall environmental sensitivity: some adolescents are more sensitive to environmental influences in general (e.g., sensory stimuli) than other adolescents. They may also be more strongly affected by the perception of subtle day-to-day changes in parenting. In sum, adolescents who appraise and perceive parental behavior as more need-supportive, should benefit more in terms of their well-being^2^. Therefore, in line with this theoretical reasoning^2,25^, but also correlational^26^ and experimental work^27^, we hypothesize that child-reported parenting (as it also encompasses adolescents’ perception and appraisal) should be more strongly related to well-being than parent-reported parenting.

### A new methodological approach

To date, most research testing the universality of associations between need-supportive parenting and adolescents’ well-being has compared cross-sectional associations across different cultures^6,28^ and across different personality traits^7^. Evidence for similar positive associations between parenting and adolescent well-being across cultures and personality is seen as confirmation of the notion that need-supportive parenting is beneficial for every adolescent’s well-being.

However, an absence of differences between groups in an average effect does not imply that (within groups) each adolescent is affected positively (one size fits all fallacy)^19,20,29^. In other words, evidence that the average adolescent in a group feels better when their parent is autonomy-supportive does not automatically imply that this is true in *every* family of which the group is composed. In fact, scholars adopting a relativistic perspective would argue that such an average effect does not need to be true for any family^30^.

Leading methodologists^31–34^ therefore, advocate that each unit within a group (e.g., the individual person or the individual family) should be studied in-depth. Here we adopt such a family-specific approach as a powerful test whether theoretically assumed parenting processes are uniform, universal, or unique. To establish family-specific effects, we assessed each family very frequently (up to 100 times), using a daily diary approach^35^, in which participants answer a questionnaire at the end of each day for more than three months.

Assessing the average within-family parenting effect, previous daily diary research^17,20,36^ confirms that on days when parents are warmer and more autonomy-supportive than usual, the average child also reported feeling better (compared to other days). Testing whether these effects are uniform, a number of recent intensive longitudinal studies with up to 18 repeated measurement^19,20^ found that the associations between parental warmth and affect were *not uniform* but differed widely between families, even within homogeneous subgroups^20^. But does this variance in parenting effects point at the direction of truly unique or at universal mechanisms (see Fig. 1)? This critical next step can only be taken when a study is sufficiently powered to assess this question at the level of an individual family.

### The current study

This study thus aims to address a fundamental, but unanswered, question in the psychological literature: to which extent is our everyday functioning driven by *uniform, universal*, or *unique* mechanisms? SDT would argue two key dimensions of need-supportive parenting (i.e., parental warmth and autonomy support) are fundamental ingredients for every adolescent and should therefore be universally positively linked to their well-being. To date, a rigorous test that each adolescent benefits from this type of parenting is still missing: Are there really (nearly) no families contradicting these theoretical predictions?

Our 100-day diary study with both child- and parent-reported parenting, which is five times longer than a typical diary study in our field (*M* = 18 days, Range = 4–56)^36^, allowed us to assess, for each family, whether the hypothesized within-family parenting associations would apply. Based on the SDT perspective on parenting, we predicted *universalism without uniformity*^2,5,21^. With regard to how daily parenting (parental warmth and autonomy support) influences adolescents’ daily affective well-being (high levels of positive and low levels of negative affect), we predicted (for preregistration, see: https://osf.io/j26k8):

**H1:** Associations are *not uniform* but differ in strength between families

**H2:** Associations are *universal* (i.e., in (almost) no families opposing effects are found).

**H3**: Associations with child-reported parenting are stronger than with parent-reported parenting.

## Results

### Descriptive analysis

As indicated by the intraclass correlation coefficient (ICC; Table 1), up to 100 days of data per family indicated that parenting and well-being varied from day to day. Between 35 and 53% of the variance in the measures was due to such within-family (daily) fluctuations (1-ICC). Whether these over time fluctuations in how parents behave and how adolescents feel are meaningfully associated was subsequently examined at the level of individuals families (average associations can be found in Table 1).

**Table 1:** Descriptive statistics and correlations for study variables.

	M	SD	ICC	1	2	3	4	5	6
**Child report**									
1 Parental warmth	83.31	17.39	0.56	–	0.39*	0.33*	− 0.23*	0.23*	0.08*
2 Autonomy support	74.57	24.97	0.59	0.68*	–	0.20*	− 0.14*	0.13*	0.10*
3 Positive affect	76.49	20.68	0.62	0.51*	0.42*	–	− 0.50*	0.10*	0.07*
4 Negative affect	10.99	14.94	0.47	− 0.37*	− 0.29*	− 0.65*	–	− 0.09*	− 0.06*
**Parent report**									
5 Parental warmth	79.99	14.91	0.65	0.41*	0.30*	0.09	− 0.04	–	0.30*
6 Autonomy support	74.70	17.38	0.52	0.31*	0.34*	0.06	0.02	0.83*	–

ICC, intraclass correlation coefficient. All items ranged from 0 to 100. Between-family correlations are presented under the diagonal (and represent associations between average levels), within-family correlations are presented above the diagonal (and represent how day-to-day fluctuations are associated) * *p* < 0.001.

### Dynamic structural equation models

#### Average associations

Estimates of the four preregistered dynamic structural equation models (DSEM; parenting [parental warmth & autonomy support] × affect [positive & negative])^37^ with the child-reported data are displayed in Table 2. DSEM modelling combines *n* = 1 time-series analyses, with Bayesian multilevel models, and Structural Equation Modeling (SEM). This allows to simultaneously analyze how parenting and affect were associated within each family (co-fluctuations from day to day) as well as on the between-family level (comparing averages between families). In the average family, on days when adolescents reported higher parental warmth or higher autonomy support (compared to other days), they also reported higher average levels of positive and lower negative affect (PA: β = 0.18–0.30; NA: β = − 0.21 to − 0.13). Similarly, comparing families amongst each other, adolescents who reported on average more parental warmth or autonomy support (compared to other adolescents) across the 100 days reported more overall positive and less negative affect (PA: *r* = 0.48–0.58; NA: *r* = − 0.38 to − 0.30).

**Table 2:** Model results of dynamic structure equation models (child reported need-supportive parenting).

	Positive affect			Negative affect		
	Est.	Est. St.	95% CI	Est.	Est. St.	95% CI
**Parental warmth**						
Average within-family						
Parental warmth (t)→Affect (t)	0.34	0.30	**[0.29; 0.38]**	− 0.20	− 0.21	**[**− **0.24; **− **0.17]**
Affect (t−1)→Affect (t)	0.26	0.26	**[0.23; 0.30]**	0.24	0.24	**[0.20; 0.27]**
Average between-family						
Parental warmth & Affect	121.84	0.58	**[86.61; 158.58]**	− 51.85	− 0.38	**[**− **79.19; -29.63]**
Variance within-family						
Parental Warmth (t)→Affect (t)	0.05		[0.03; 0.07]	0.03		[0.02; 0.05]
Affect (t−1)→Affect (t)	0.03		[0.02; 0.04]	0.02		[0.02; 0.03]
Ratio: SD/fixed effect (H1)						
Parental warmth (t)→Affect (t)	**0.65**		[0.54; 0.80]	**0.89**		[0.72; 1.16]
Affect (t−1)→Affect (t)	**0.66**		[0.55; 0.82]	**0.65**		[0.52; 0.83]
**Autonomy support**						
Average Within-family						
Autonomy support (t)→Affect (t)	0.15	0.18	**[0.12; 0.18]**	− 0.09	− 0.13	**[**− **0.11; **− **0.07]**
Affect (t−1)→Affect (t)	0.30	0.30	**[0.26; 0.33]**	0.25	0.25	**[0.21; 0.29]**
Average Between-family						
Autonomy support & Affect	147.84	0.48	**[97.02; 210.11]**	− 60.12	− 0.30	**[**− **98.56; **− **28.82]**
Variance within family						
Autonomy support (t)→Affect (t)	0.02		[0.02; 0.03]	0.01		[0.01; 0.02]
Affect (t−1)→Affect (t)	0.03		[0.03; 0.05]	0.03		[0.02; 0.04]
Ratio: SD/Fixed Effect (H1)						
Autonomy support (t)→Affect (t)	**1.02**		[0.82; 1.33]	**1.28**		[0.98; 1.81]
Affect (t−1)→Affect (t)	**0.62**		[0.52; 0.75]	**0.65**		[0.53; 0.83]

Bold values indicate significant/meaningful estimates.

Est., unstandardized estimates; Est. St., standardized estimates for fixed within- and between-family effects, standardized using the STDYX Standardization (Within-Level Standardized Estimates Averaged over Clusters) in M*plus*; Ratio, Random Slope SD/Fixed Effect: a point estimate > 0.25 is the criterium we defined as meaningful effect heterogeneity^31^; 95% CI, 95% Credibility interval.

#### Non-Uniformity of parenting-affect associations (H1)

To move beyond averages and to test if parenting associations might be uniform, we first inspected per individual family how parenting and adolescents’ affect were associated (i.e., family-specific effects). Our first hypothesis that parenting-affect associations would not be uniform was supported: In all four models the standard deviation (indicating how much families differ) was more than 25% of the average effect in families^31^ (Ratios: 0.65–1.28, see Table 2). This suggests that families differ meaningfully in how parenting processes function within-families.

#### Universality of parenting-affect associations (H2)

**Figure 2:**
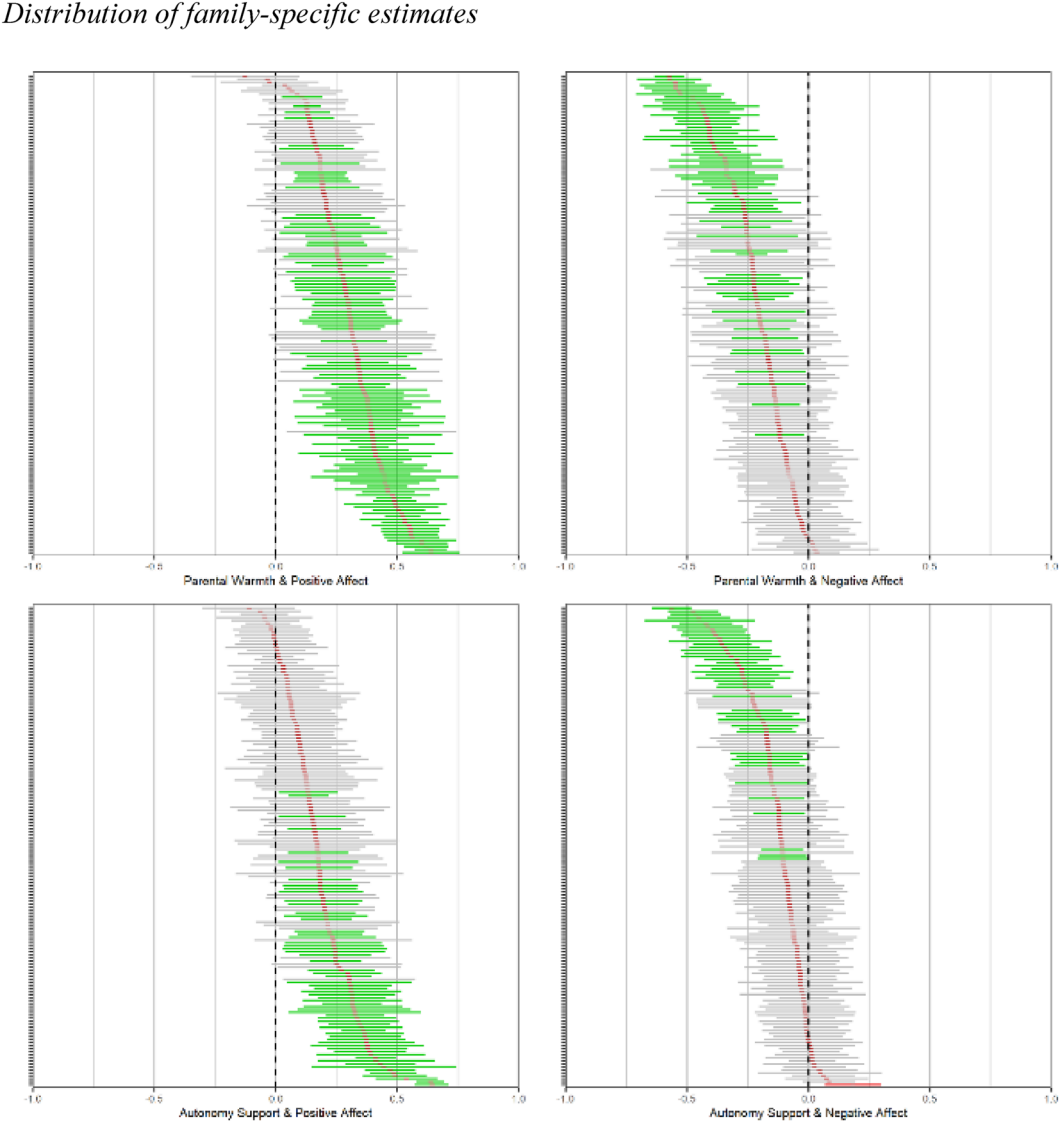
Note: Red points indicate the estimated effect per family, bar indicates an approximation of the credibility interval. The color of the bar indicates the ‘classification’ of this family. Green, correctly classified; grey, ambiguously classified; red, incorrectly classified.

Second, we tested whether associations between parenting and adolescent affect would be universal. In other words, they should not be identical in each family, but there should hardly be any family in which opposing patterns are found. In 91–98% of the families, the estimated effects of parenting upon adolescent well-being were in the expected direction (see Table 3 and Fig. 2). As preregistered, we ran a rigorous test of our hypothesis for each model, by categorizing families in three groups based on whether the effects were significant at the family-specific (*N* = 1) level and follow the theoretically predicted pattern^34^ (see Table 3): ‘correctly classified’ (significant family-specific association in the expected direction; 29–66%), ‘ambiguously classified’ (non-significant family-specific association; 34–70%), and ‘incorrectly classified’ (significant family-specific association in the unexpected direction; 0–1%).

**Table 3:** Direction of point estimates and ‘classification’ of family-specific estimates in the models with child-reported parenting (H2).

Model	Expected direction of effect	Direction of effect		‘Classification’		
		Correct *N* (%)	Incorrect *N* (%)	Correct *N* (%)	Ambiguous *N* (%)	Incorrect *N* (%)
Parental warmth & positive affect	Positive	156 (98%)	3 (2%)	105 (66%)	54 (34%)	0 (0%)
Parental warmth & negative affect	Negative	154 (97%)	5 (3%)	65 (41%)	94 (59%)	0 (0%)
Autonomy support & positive affect	Positive	146 (92%)	13 (8%)	63 (40%)	96 (60%)	0 (0%)
Autonomy support & negative affect	Negative	144 (91%)	15 (9%)	46 (29%)	112 (70%)	1 (1%)

For 20% of the families, each of the four family-specific parenting-affect associations was correctly classified.

**Figure 3:**
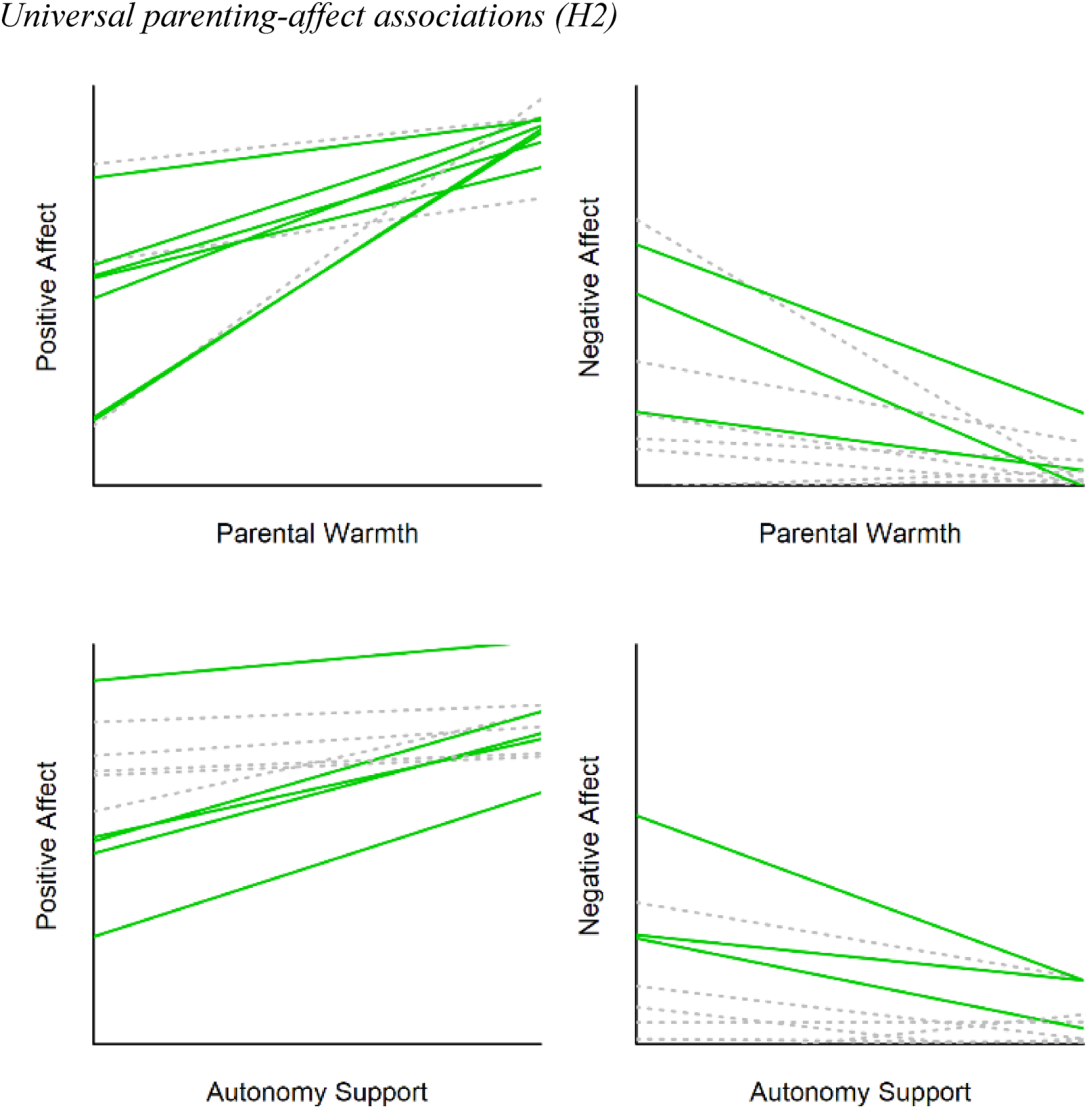
Note: Each line represents the association between parenting and well-being for one family. Ten exemplary families are depicted per model. Green solid lines indicate a significant association in the expected direction (‘correctly classified’) and grey dotted lines indicate a non-significant association (‘ambiguously classified’).

Looking at the overall evidence across models (Table 3 and Fig. 3), we estimated 636 parenting effects (159 families × 4 models), of which 279 (44%) were significant and in the theoretically expected direction. Only one effect for one single individual (0.2%) contradicted the theoretical hypotheses (see supplement for inspection of data). Hence all preregistered criteria for the universality hypothesis (H2) were met. Firstly, we found only 0.2% incorrectly classified families (criterion: < 5%), which showed an unexpected pattern. Secondly 279 times more families were correctly classified than incorrectly classified (criterion: 3 times). Thirdly, we found 44% significant results at the *N* = 1 level (criterion: ≥ 10%). Overall, our findings show that, even though parenting-affect associations differed in terms of their strength (H1), hardly any family showed a theoretically unexpected pattern (H2). Together this provides very strong evidence for the universality hypothesis.

#### Perceptions of parenting (H3)

Additionally, we assessed whether adolescents’ perceptions of parenting would be more strongly related to their affective well-being than their parents’ perception (H3) by replacing child-reported by parent-reported parental warmth and autonomy support. Results of these four models (see supplemental materials) provide additional support for H1 and H2: also when using parent reports for parenting the association between parenting and adolescent affect differed in size but not direction of effects (17% correctly classified, versus 0.2% incorrectly classified). Moreover, as expected (H3), adolescents’ own perspective on parenting were related more strongly to their affective well-being than their parents’ perspective on parenting (*d* = 0.56–1.46). As shown in Table 4, when family-specific estimates from the child-reported models and parent-reported models were compared with matched t-tests (see Table 4) all four comparisons supported Hypothesis 3.

**Table 4:** Differences in family-specific estimates between adolescents’ and parents’ reported parenting (H3).

Model	Adolescent		Parent		Respondent difference				
	*M*	*SD*	*M*	*SD*	*t*	*df*	*p*	*d*	95% CI
Parental warmth & positive affect	0.31	0.14	0.13	0.10	15.21	158	< 0.001	1.46	[1.21, 1.71]
Parental warmth & negative affect	− 0.22	0.14	− 0.11	0.14	9.23	158	< 0.001	0.74	[0.51, 0.96]
Autonomy support & positive affect	0.18	0.14	0.07	0.09	10.32	158	< 0.001	1.01	[0.77, 1.24]
Autonomy support & negative affect	− 0.13	0.13	− 0.07	0.09	6.20	158	< 0.001	0.56	[0.34, 0.79]

95% CI = 95% confidence interval.

#### Sensitivity analysis

Several preregistered sensitivity analyses were conducted to assess the robustness of the findings. The universality without uniformity principle (H1 & H2) was supported in most additional models (90%), in which we (1) doubled the number of iterations to estimate the model, (2) excluded participants with few datapoints, (3) used parent-reported parenting, (4) assessed lagged effects, (5) did not control for prior day affect, (6) and varied the number of participants numbers of assessments (*t* = 10, *t* = 25, *t* = 50, *t* = 75) (for a detailed description: see supplemental materials).

#### Exploring moderators to explain individual differences

To understand why the effects of parenting within families may be different, we correlated the family-specific results from the child-reported models with theoretically relevant moderators (see Table 5). Adolescent demographics (1/12 tests significant), adolescent personality (3/20 tests significant), and level of parenting (2/8 tests significant) were mostly unrelated to the strength of associations. However, among adolescents with higher overall levels of environmental sensitivity parental warmth and autonomy support were more strongly tied to their positive and negative well-being – and this finding was consistent across four models.

**Table 5:** Correlates of between-family differences in family-specific parenting-affect associations.

	Parental warmth Positive affect	Parental warmth Negative affect	Autonomy support Positive affect	Autonomy support Negative affect
**Adolescent demographics**				
Age	0.09	0.04	0.03	− 0.01
Gender	0.10	− 0.09	**0.26**	− 0.20
Education	− 0.03	− 0.14	− 0.11	− 0.04
**Adolescent traits**				
Neuroticism	0.02	− 0.15	**0.27**	− **0.29**
Extraversion	− 0.05	0.01	− 0.06	− 0.01
Openness	0.14	− 0.20	0.09	− **0.21**
Agreeableness	0.04	− 0.07	0.03	0.01
Conscientiousness	0.11	− 0.06	0.02	− 0.00
Environmental Sensitivity	**0.30**	− **0.32**	**0.38**	− **0.35**
**Average levels of parenting**				
Average levels of warmth	0.13	− 0.18	− **0.22**	0.19
Average levels of autonomy support	0.05	− **0.20**	− 0.10	− 0.02

signfiicant values are in bold (Credibility interval does not contain 0).

Gender was coded 0 = male, 1 = female.

## Discussion

Whereas some theoretical paradigms in psychology stress that each person is unique and follows the logic of an unpredictable dynamic system^38,39^, other theories stress that some of the mechanisms which drive and determine our thoughts, behaviors and feelings may still hold for each of us^4^. This ongoing debate with regard to this universality versus uniqueness of human functioning, is also clearly visible when it comes to understanding how parenting promotes adolescents’ well-being^2,3^. Self-Determination Theory (SDT) would assume that parental warmth and autonomy support are universally beneficial for adolescents’ well-being because these parenting dimensions support adolescents’ basic psychological needs^5,12^. At the same time, this theory recognizes that adolescents might benefit to a different degree from need-supportive parenting^2,21^. One of the consequences of this hypothesis, is that it assumes no child should respond to need-supportive parenting with poorer well-being.

The current study tested this assumption of ‘universality without uniformity’^2^ by applying a novel family-specific approach to 159 families. Each of them was followed for 100 days. As such, we could demonstrate that need-supportive parenting was linked to better adolescent well-being in 91–98% of the families. And although somewhat different in strength, less than 1% of the families displayed an unexpected pattern (H1 & H2). Furthermore, as predicted, adolescents’ own perspective on parenting was more strongly related to their affective well-being than parents view on how need-supportive they were (H3). The extent to which adolescents responded to parenting was partially explained by their stable traits: Adolescents who were more sensitive to environmental influences in general also benefited more from need-supportive parenting in daily life.

### Universality without uniformity

One of the questions many parents have is how to safely navigate their children through adolescence. When are they doing the right thing? And which advice would apply to *their own* child? Theoretically, parental warmth and autonomy support are assumed to universally satisfy adolescents’ basic psychological needs, and as such promote subjective well-being for all adolescents^5^. Indeed, in all four preregistered confirmatory models (2 parenting × 2 affect) the way in which parenting related to adolescent’s well-being differed in size but not direction of effects. From all 636 family-specific associations, only one contradicted the theoretically assumed predictions (0.2%), and we found 279 times more support for the benefits of need-supportive parenting than evidence against it. Together with several exploratory models (e.g., parent-reported models) this study provided strong and consistent evidence that SDT’s *universality without uniformity* principle applies to the everyday lives and interactions of adolescents and their parents. Contrasting relativistic accounts on parenting^3^ which suggest parenting effects may be unique for each child, these results stress that need-supportive parenting may be a universal ingredient for parents to promote their adolescent’s everyday well-being.

When comparing the positive versus negative dimension of subjective well-being, need-supportive parenting seemed more beneficial for adolescents’ positive affect. This finding is in line with the dual pathway perspective on the basic psychological needs. Need-supportive contexts are assumed to play a stronger role in positive developmental outcomes (i.e., the bright pathway) and where need-thwarting contexts would play a stronger role in negative outcomes (i.e., the dark pathway)^17,40^. Future research which also includes such need-frustrating behaviors, such as psychological control^5,17^ are therefore needed to establish whether this universality also holds for parenting practices which are potentially harmful.

Moreover, this study specifically focused on understanding how parenting, an important developmental context, may influence an adolescents’ well-being in the everyday lives. Even though strong linkages were found in some families, everyday parenting effects were quite small in others. However, when considering development as a dynamic system^38^, in which everyday interaction may carve out a more stable pattern of functioning, these may still be meaningful. Additionally, small effects may also point to a more stable state of functioning, in which contextual changes no longer impact the child. According to SDT, for instance, adolescents may get desensitized to need-supportive contexts after a longer developmental history of need-thwarting experiences^41,42^, such that adolescents no longer respond to new opportunities for need satisfaction. As such, to assess how the everyday interactions with parents ultimately determine an adolescents’ developmental course, and vice versa, future research would do well to examine adolescents’ sensitivity to need-supportive parenting not only at the everyday timescale at which interactions take place, but also across longer periods of time.

### Exploring differences between families

SDT offers several explanations for the fact that children do not uniformly benefit. In understanding non-uniformity, adolescents’ perspectives on parenting were a key factor. The current study found that adolescents’ perception of daily parenting was more predictive of their affective well-being than parents’ perception thereof. This is consistent with the notion that children’s perceptions of parental behavior (rather than actual parental behaviors or parental intentions) ultimately determine children’s responses to parenting and their subsequent adjustment^43^. Still, these results do not suggest that it is just a matter of perception, and it is completely trivial what parents are doing. Parents’ reports of daily parenting also were found to be universally beneficial to adolescents’ daily affective well-being—this finding is helpful in future translational work towards parenting interventions.

Possible reasons why adolescents perceive and/or appraise their environment differently could lie in trait-level differences. Our results confirmed environmental sensitivity theory^23,24^, showing that adolescents who score higher on environmental sensitivity, or related constructs (like neuroticism^24,44^), are not only more sensitive to sensory stimuli (e.g., smells or sounds) but seem also more reactive to need-supportive parenting.

### Implications and limitations

Answering questions with regard to the universality and uniqueness of human development and functioning requires a new type of research designs, which move beyond the group average. Capitalizing on possibilities to use smartphone technology and data collection software, investing in the gamification of research participation^45^, and using new analytical techniques for time series data^37^, this unique study has shown for the first time that assessing up to 100 measurements per family is feasible. To the best of our knowledge, this is the longest daily diary study in parenting research ever conducted^36^. Such a design allows to study everyday functioning in each individual and answers fundamental questions about child development. However, being limited by the number of items in our daily questionnaires, it is an open empirical question if these universal effects also apply to other parenting dimensions (e.g., psychological control) or even other environmental factors (e.g., social media)^46^.We therefore reiterate the call of methodologists^31–34^ to leverage the current family-specific approach to a wide range of contextual influences (such as friendships, work/school, or social media) and related research questions. This has the potential to unravel the extent of universality and uniqueness of human development and functioning.

With regard to the here studied associations, discovering universal parenting-affect associations is important not only from a fundamental perspective but also from an applied perspective. In practice, ideally, parenting interventions are as universal as possible, but as tailored as needed. Based on the here detected universal associations, promoting need-supportive parenting advice or parenting interventions^47,48^ appears quite justified as every child should profit (to a certain amount) when their parent adopts more need-supportive parenting. However, tailored parenting advice might be needed, as children differ in their sensitivity to need-supportive parenting. Parenting advice should address that need supportive-parenting must be provided in a way that matches the child’s unique needs and characteristics^48^. Such a parenting advice that applies universal perspectives yet considers the specific features of individual families^48^, would be really putting the universality without uniformity principle into practice.

However, before such implications can be justified, this novel work calls for rigorous replications, which could also address some of the current shortcomings. Firstly, although our study was powered to detect effects that are typically considered small to medium with 100 datapoints per family (see power analysis), we were still limited in our ability to pick up even smaller family-specific effects. Indeed, the distribution of family-specific effects (see Fig. 2) indicated that there were positive associations between need-supportive parenting and affect among virtually all families (91–98%) even though only a subgroup (29–66%) of effects reached significance. Future research could further increase statistical power by for example including more assessments per family. Secondly, the generalizability of these findings needs to be assessed. It would be valuable (1) to include a more diverse sample in terms of age, socio-economic status, cultural and ethnical background, (2) to assess several socialization figures (e.g., other parents or teachers) and (3) to include measures to study the mediating pathway of need-satisfaction to better understand the mechanism between parenting and adolescent well-being. Thirdly, with assessing *daily* effects, the current study cannot make claims about long-term benefits of need-supportive parenting. It is possible that all adolescents enjoy days when their parents are warm and autonomy-supportive but that not all of them will eventually be happier adults. It is an empirical question, whether these universal short-term effects translate to universal longer-term benefits for adolescents’ well-being (galloping horse fallacy)^29^.

## Conclusion

The ongoing theoretical debate about the universality versus the uniqueness of human functioning and adaptation^21,39^, is also clearly visible in the parenting literature^2,3^. Whereas some theories stress that the effects of parenting highly depend on the child, the parent and the environment^3,38,39^ others would argue that universal ingredients exist^2,5^. The current preregistered 100-day diary study among 159 families tested if parenting was uniformly, universally, or uniquely linked to child affective well-being, by using a family-specific paradigm. For each family, we assessed whether and how two dimensions of parenting, which are considered universally beneficial (parental warmth and autonomy support) would predict adolescent affective well-being. Whereas 279 estimates supported this hypothesis, only 1 estimate was significant in the unexpected direction. This study, as such provides robust evidence for the *universalism without uniformity* principle^2^: Parental warmth and autonomy support might benefit adolescents’ daily well-being in (almost) all families—and may as such be one of the universal ingredients for parenting teens.

## Method

### Sample

In the Dutch “100 days of my life” study (https://osf.io/5mhgk/), 159 parent-adolescent dyads took part. Adolescents were on average 13.31 years old (*SD*_age_ = 1.22, Range_age_ = 12–16), and more girls (62%), than boys (36%) participated. Some did not identify with either being male or female (2%). Adolescents had different backgrounds. Most adolescents (89%) were born in the Netherlands, and others in other European countries (6%), Asia (2%), North America (1%), South America (1%), or Africa (1%). Adolescents followed different educational tracks: a higher educational track (51% pre-university secondary education), a medium educational track (30% higher general secondary education), a lower educational track (15% pre-vocational secondary education and vocational training) or a mixed educational track (5%).

Participating parents were the biological mother (79%), the biological father of the participating adolescent (19%), or another caregiver (*n* = 1 adoption mother, *n* = 1 second mother, *n* = 1 stepfather). Parents were on average 45.34 years old (*SD*_age_ = 4.54, Range_age_ = 33–55). In terms of educational level, 62% of the parents reported higher education (college or university degree), 25% medium education (vocational/technical training), and 10% of the parents reported lower education (high school diploma). The remaining 3% gave insufficient information to classify them. Parents (87%) were born in the Netherlands, or elsewhere (6% other European countries, 3% Asia, 1% North America, 1% South America, 1% Africa, 1% Australia).

With regard to family compositions, most parent-adolescent dyads lived together (81%), others only lived with the participating parent some days of the month (18%; *M* = 18 days per month, *SD* = 4.68; Range = 5–27). One participant indicated not living together with the parents (but did have regular contact with them). Most adolescents had contact with their biological parents (92%), others only with their biological mother (8%). One out of ten adolescents had stepparents. One participant had an adoption mother and adoption father, and another participant had two mothers. Adolescents on average had one sibling (Range = 0–5).

### Procedure

Most families were informed about the study via two high schools in the Netherlands, with each school consisting of about 2,000 students from all educational tracks. These families were contacted by mail, social media, posters, and class visits. Other families heard about the study via the research team by personal communication, social media, and a newsletter to former participants. After a detailed briefing via a video call, interested families signed an online informed consent form and received information on how to install the app. One adolescent (12–16 years) and one parent per family were allowed to participate if they had contact with each other nearly every day and both owned a smartphone.

For 100 consecutive days (Oct 26, 2020 until Feb 2, 2021), both adolescents and parents answered one daily questionnaire containing 24–28 items (approx. 3–5 min) via the Ethica Data app^49^ on their own smartphone. Both iOS (61%) and Android (39%) operating systems were used. The questionnaires were available from 5PM until 12PM the next day, and participants were prompted in the evening, depending on their own preference (7PM, 8PM, 9PM or 10PM). After the initial prompt, participants received a maximum of four automatic reminders every 30 min and one at 7 AM the next morning. After the 100 days, participants could choose to catch up missed questionnaires by extending their participation period up to 25 days. At the start of the 100 days (‘baseline’) participants answered additional online questionnaires (ca. 30–45 min) about their traits (e.g., Big Five personality and environmental sensitivity).

Participants received a monetary reward for each answered questionnaire and a bonus if they answered 100 daily questionnaires and/or answered 10 questionnaires in a row. Adolescents could receive up to €100 (≈ US$ 113) and parents €50 (≈ US$ 57). Every day 2×€10 were raffled among adolescents who answered the daily questionnaire. This study was approved by the Ethical Committee of Tilburg University (RP250) and all methods were performed in accordance with the relevant guidelines and regulations. More information about the procedure can be found on OSF (https://osf.io/5mhgk/).

Adolescents answered on average 93 daily questionnaires (range 24–108; total 14,797). On 91 days adolescents also reported to have seen their participating parent (range 24–108, total 14,546). Parents answered on average 97 daily questionnaires (range 21–120; total 15,372) and on 96 days parents reported to have seen their participating adolescent (range 20–120, total 15,201).

## Materials

### Daily measures

All daily diary items were scored on a visual analogue scale ranging from 0 (*Not at all*) to 100 (*Very much*). Visual analogue scales show equal psychometric qualities as Likert scales and high school students prefer them above Likert scales^50,51^.

#### Daily parental warmth

Both adolescents and parents rated two items daily about their daily parental warmth. The items were: “The relationship with my mother/father was enjoyable” and “My mother/father showed me that she/he cares for me.” These two items tap into the two main components of parental relational support, that is, (a) the provision of affection and (b) parental care and responsiveness^5,16^. Parents received parallel questions (e.g., “I showed my child that I cared for him/her.”). The items were adapted from a Dutch daily diary study (Research on Adolescent Development and Relationships [RADAR])^52^ which were based on the Network of Relationships Inventory (NRI)^53^. Both items correlated strongly at the within-family level (adolescent: *r* = 0.64; parent: *r* = 0.56) as well as at the between family-level (adolescent: *r* = 0.85; parent: *r* = 0.90).

#### Daily parental autonomy support

Both adolescents and parents rated two items daily about their daily parental autonomy support. The items were: “My mother/father allowed me to make my own plans.” and “My mother/father took my point of view into account.”. These items aim to capture the two main components of parental autonomy support, that is, (a) the provision of choice and allowance of independent decision-making and (b) acknowledgment and interest in the adolescents’ perspective^5,6^. Parents received parallel questions. The items were adapted from a Dutch daily autonomy support scale^17^ which consisted of 4 items and was based on the Perception of Parents Scale (POPS)^54^. The items correlated moderately strong at the within-family level (adolescent: *r* = 0.46; parent: *r* = 0.34) and strongly at the between-family level (adolescent: *r* = 0.76; parent: *r* = 0.70).

#### Daily positive and negative affect

Adolescents rated daily their positive affect (“joyful” and “happy”) and negative affect (“mad”, “afraid”, and “sad”). These scales are a shortened version of the Positive and Negative Affect Schedule for Children (PANAS-C)^9^. The current items were chosen, based on previous work on psychometric properties of the Dutch scale^19,55^ and theoretical considerations to assess the basic emotions (happiness, anger, anxiety & sadness). The items for positive affect correlated highly at the within- and between-family level (within: *r* = 0.76; between: *r* = 0.95). The scale for negative affect also showed good internal consistency at the within- and between-family level (ω_within_ = 0.71; ω_between_ = 0.92).

### Baseline measures

#### Big Five personality

Adolescents answered the short version in easy language of the Dutch Big Five Inventory-2^56^ during the baseline assessment. They answered 30 items on a 5-point Likert scale (1 “totally disagree” to 5 “totally agree”). Five subscales were calculated: Neuroticism (e.g., “I worry a lot.”), Extraversion (e.g., “I am outgoing and sociable.”), Openness (e.g., “I am fascinated by art, music or literature.”), Agreeableness (e.g., “I am compassionate and have a soft heart.”) and Conscientiousness (e.g., “I keep things need and tidy.”). The scales’ internal consistencies ranged from ω_between_ = 0.65–0.80.

#### Environmental sensitivity

Adolescents answered the Short Version of the Dutch Hypersensitivity Child Scale (HCS)^44^ during the baseline assessment. They answered 12 items on a 7-point Likert scale (1 “Not at all” to 7 “Extreme”). An example item is: “I notice when small things have changed in my environment.”. The scale showed good internal consistency (ω_between_ = 0.74).

### Preregistered analysis plan

The analysis plan and hypotheses were preregistered before the data were accessed (see: https://osf.io/j26k8). Dynamic Structural Equation Modelling (DSEM) was used in this study, which combines Bayesian multilevel modeling with n = 1 time-series modeling and structural equation modelling^37^. The strength of this unified approach is that the model uses all information from all families to obtain more stable family-specific estimates (i.e., one estimate per individual family). Together this allows to assess in how many families parental warmth and autonomy support are related to affective well-being in theoretically predicted ways.

As time (i.e., days in the study) explained less than 1% of the variance in multi-level regression models, and visual inspection did not reveal nonstationary patterns, we assumed stationarity. The DSEM models were specified as bivariate multilevel autoregressive models (ML-AR) separately in 8 models (2 (parenting) × 2 (affect) × 2 (respondent)). The model contained two distinct levels of analyses. Firstly, at the within-family level, affect was predicted by previous day affect and same day parenting. We chose to statistically control for previous day affect to account for the temporal dependencies in the diary data. Effects at the within-family level were specified as random effects, allowing a different estimated effect per family. Secondly, at the between-family level, we examined variance around the within-family effect, and we added an association between stable levels of parenting and affect. Moreover, this is the level where we added moderators, to understand whether stable differences between individuals (e.g., in their personality) can explain different effects within families.

We conclude that parenting-affect associations are *not uniform* but differ in size (H1) if the between-person variance around family-specific estimates is meaningful (i.e., the standard deviation of the random effect is at least 25% of the absolute value of the fixed effect)^31^. Inspecting family-specific estimates, as well as their family specific significance, we conclude that parenting-affect associations are *universal* (H2), if three preregistered conditions are met: (a) < 5% of participants show statistically significant family-specific effects that contradict the theory, (b) at least three times more participants show significant family-specific effects in line with theory, and (c) ≥ 10% of participants have significant family-specific effects. Finally, we can conclude that adolescents’ perception of parenting is more closely related to their affect (H3) if family-specific estimates of child-reported models are significantly stronger (matched t-tests with an alpha level of 5%) than family-specific estimates of the parent-reported models.

### Power analysis

One unique feature of the current study is that we estimated family-specific effects (H2). Power for our analysis could not be derived analytically. We therefore approximated the power to detect significant effects for a single family post-hoc, using a multiple regression framework with two predictors in G*Power, we estimated that with the average number of observations (*t* = 91), we could detect with 80% power an effect that explains 8% variance (β ≈ 0.28). Smaller effects that explain 5% (β ≈ 0.22) or 1% of the variance (β ≈ 0.10) could only be detected with 58% and 16% power, respectively. When we compare family-specific effects from child-reported models and parent-reported models (H3), analyses in G*Power indicate that these matched t-tests can detect small effects (*d* = 0.22) with 80% power. For all power analyses, we applied an α-level of 5% (two-tailed).

## Supplementary Information

Supplementary Information 1.

Supplementary Information 2.

Supplementary Information 3.

## Data Availability

The preregistered analytical plan (https://osf.io/j26k8), codebook of the study (https://osf.io/5mhgk/), and supplemental materials (https://osf.io/4cy87/) are shared on OSF. The aggregated datasets analysed during the current study are available on OSF, https://osf.io/kqg92/; The raw datasets are available from the corresponding author on reasonable request.
