# Supplementary material for: Universal ingredients to parenting teens: parental warmth and autonomy support promote adolescent well-being in most families
Source: Sci Rep. 2022 Oct 7;12:16836. doi: 10.1038/s41598-022-21071-0 (PMC9546835; doi:10.1038/s41598-022-21071-0)
Supplement: Supplementary file 2 — Supplementary Information 2. [file 41598_2022_21071_MOESM2_ESM.docx]

Supplemental Materials for

**Universal Ingredients to Parenting Teens: Parental Warmth and Autonomy Support Promote Adolescent Well-being in Most Families**

Anne Bülow^1^*, Andreas B. Neubauer^2^, Bart Soenens^3^, Savannah Boele^1^, Jaap J. A. Denissen^4^ & Loes Keijsers^1^

^1^Department of Psychology, Education & Child Studies, Erasmus University Rotterdam

^2^DIPF | Leibniz Institute for Research and Information in Education

^3^Department of Developmental, Personality and Social Psychology, Ghent University

^4^Department of Developmental Psychology, Utrecht University

* Corresponding author

**Author Note**

Anne Bülow <https://orcid.org/0000-0003-3335-7447>

Andreas B. Neubauer <https://orcid.org/0000-0003-0515-1126>

Bart Soenens <https://orcid.org/0000-0003-1581-3656>

Savannah Boele <https://orcid.org/0000-0003-2821-1312>

Jaap J. A. Denissen <https://orcid.org/0000-0002-6282-4107>

Loes Keijsers <https://orcid.org/0000-0001-8580-6000>

**Data availability and Open Practices Statement.** The preregistered analytical plan (<https://osf.io/j26k8>), codebook of the study (<https://osf.io/5mhgk/>), and supplemental materials (<https://osf.io/4cy87/>) are shared on OSF. The aggregated datasets analysed during the current study are available on OSF, <https://osf.io/kqg92/>; The raw datasets are available from the corresponding author on reasonable request.

**Disclosure of interests.** The author(s) declare no competing interests.

**Acknowledgements.** We are grateful for the participating families, and the support of Claire Laudij-van Koot, David Harris, Dani Trommelen, Manon Enting, Laura Rust, and Demi van den Ende in collecting these data. We would like to thank Dr. Leonie Vogelsmeier, Dr. Jana Vietze & Prof. Dr. Annemiek Harder for their valuable feedback on the manuscript.

**Funding.** This research was supported by a grant from the Netherlands Organization for Scientific Research (NWO-VIDI; 452-17-011) awarded to Loes Keijsers.

**Author contributions. AB**: Conceptualization, Methodology, Formal Analysis, Investigation, Data Curation, Writing – Original Draft, Visualization, Project Administration, **AN**: Conceptualization, Methodology, Writing – Review & Editing, **BS**: Conceptualization, Writing – Review & Editing, **SB**: Conceptualization, Writing – Review & Editing, **JJAD**: Conceptualization, Writing – Review & Editing, Supervision, **LK**: Conceptualization, Methodology, Writing – Review & Editing, Supervision, Funding acquisition.

**Correspondence.** Correspondence concerning this article should be addressed to Anne Bülow,

Department of Psychology, Education and Child Studies, Erasmus University Rotterdam, P.O. Box 1738, 3000 DR Rotterdam, The Netherlands, Email: [bulow@essb.eur.nl](mailto:bulow@essb.eur.nl)

# Table of Contents

[Table of Contents 2](#_Toc101784354)

[Additional Information to Method Section 3](#_Toc101784355)

[Visualization of Model Specification 3](#_Toc101784356)

[MPlus Syntax (DSEM models) 5](#_Toc101784357)

[Additional Information to Results Section 11](#_Toc101784358)

[Additional Descriptive Statistics 11](#_Toc101784359)

[Results of Parent-reported Models 12](#_Toc101784360)

[Different Visualization of Variation in Family-specific Effects 13](#_Toc101784361)

[Visualization of Association between Family-specific Effects and Environmental Sensitivity 15](#_Toc101784362)

[Sensitivity Analysis 16](#_Toc101784363)

[Exploration of Incorrectly Classified Participant 22](#_Toc101784364)

[Further Exploratory Analysis 25](#_Toc101784365)

# Additional Information to Method Section

## Visualization of Model Specification

**Figure S1**

*Model specification*


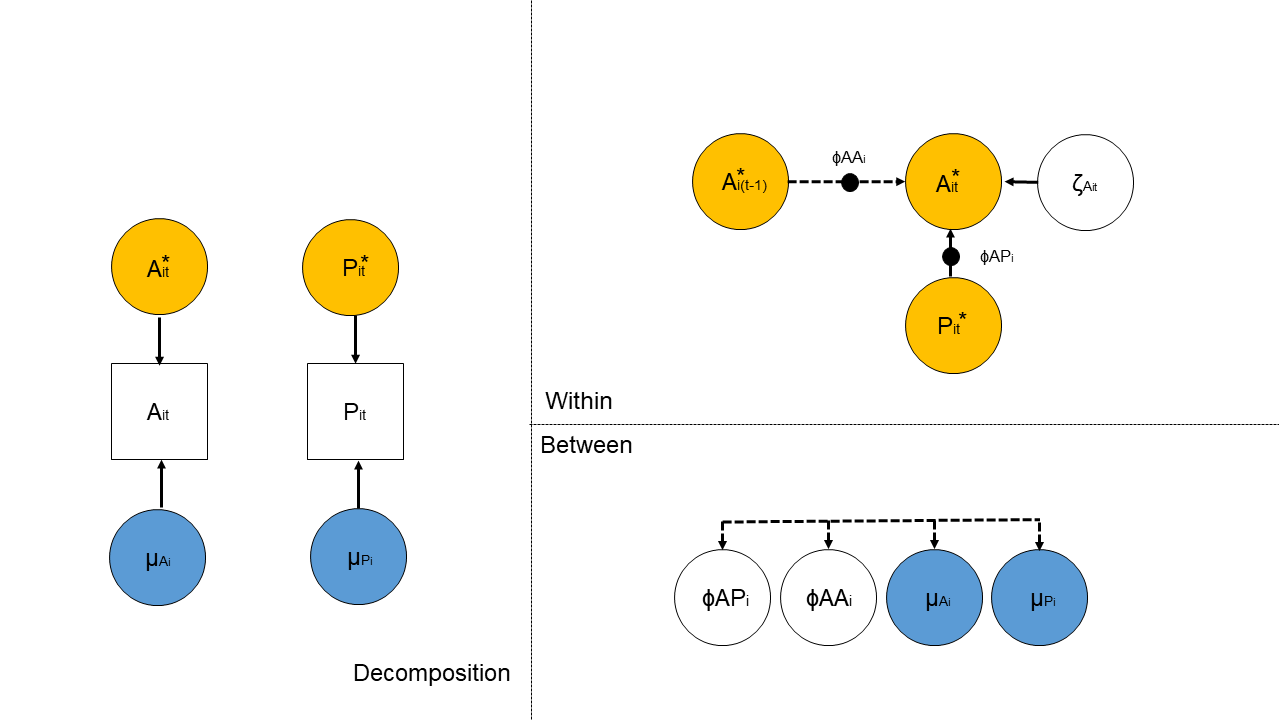
*Note*. A = affect, P = parent-adolescent interaction quality, t = time, i = participant. Left panel: Variables are decomposed in between-family part (μ = individuals mean, in blue), and within-family part (A^*^_it_ = within-family centered score of affect, P^*^_it_ = within-family centered score of parenting, in orange). Top panel: On the within-family level, affect predicts itself over time and parenting predicts affect on the same day (ϕ = regression parameter, ζ = innovation). Bullets on lines indicate estimation of random effects (i.e., family-specific effects). Bottom panel: On the between-family level, these random effects and individual means are correlated.

**Psychometric Properties of Negative Affect Scale**

**Table S1**

*Results of Multilevel confirmatory factor model for negative affect*

|  | Factor loadings | |
| --- | --- | --- |
| Item | Within-family level | Between-family level |
| Mad | .60 | .75 |
| Afraid | .57 | .91 |
| Sad | .81 | .94 |

*Note*: ω_W_ = .71; ω_B_ = .92

## MPlus Syntax (DSEM models)

In this section the M*Plus* Syntax is given, which was used to estimate the DSEMs reported in the article. Please note that next to the model which is reported in the paper (Model 1) several sensitivity analyses were conducted with slightly different specifications. These are also reported below and changes to Model 1 are highlighted in red.

**TITLE: DSEM SYNTAX FOR MODEL 1**

DATA: FILE = 'DATA.dat'; !read data

VARIABLE:

NAMES =

ID days

PAR AFF; ! Names in Dataset

! ID = ID variable

! days = day in study (1 – 125)

! PAR = Parenting variable (warmth/autonomy support)

! AFF = Positive or Negative Affect

MISSING = ALL (9999); ! Missing Values

USEVAR = PAR AFF; !Variables in this analysis

CLUSTER = ID; ! Variable which identifies participants

LAGGED = AFF(1); ! create lagged variables

TINTERVAL = days(1); !account for unequal spacing due to missing days

ANALYSIS:

TYPE = TWOLEVEL RANDOM;

ESTIMATOR=BAYES;

BITER = (5000)! Min 5000 iterations

PROC=2;

THIN = 2;

MODEL:

! Autoregressive model with time varying covariate AR(1)

%WITHIN%

!stability with lag1 (= 1 day)

aa| AFF ON AFF&1;

!hypothesized Path: Parenting predicts affect

ap| AFF ON PAR;

!Variance Affect

AFF;

!Variance Parenting

PAR;

%between%

!Intercepts

[AFF];

[PAR];

[aa] (b1);

[ap] (b2);

!Variance

AFF;

PAR;

aa (a1);

ap (a2);

!correlation between random intercepts and random slopes

aa ap PAR AFF with aa ap PAR AFF;

! Calculate the ratio SD and fixed effect (see Bolger et al)

MODEL CONSTRAINT: NEW (RATIOaa RATIOap);

RATIOaa = sqrt(a1)/b1;

RATIOap = sqrt(a2)/b2; ,

OUTPUT: TECH1 TECH8 TECH4(CLUSTER) standardized (cluster);

PLOT: type = plot1 plot2 plot3;

**TITLE: DSEM SYNTAX FOR MODEL 2**

DATA: FILE = 'DATA.dat'; !read data

VARIABLE:

NAMES =

ID days

PAR AFF; ! Names in Dataset

! ID = ID variable

! days = day in study (1 – 125)

! PAR = Parenting variable (warmth/autonomy support)

! AFF = Positive or Negative Affect

MISSING = ALL (9999); ! Missing Values

USEVAR = PAR AFF; !Variables in this analysis

CLUSTER = ID; ! Variable which identifies participants

LAGGED = AFF(1); ! create lagged variables

TINTERVAL = days(1); !account for unequal spacing due to missing days

ANALYSIS:

TYPE = TWOLEVEL RANDOM;

ESTIMATOR=BAYES;

BITER = (10000)! Min 10000 iterations for sensitivity analysis

PROC=2;

THIN = 2;

MODEL:

! Autoregressive model with time varying covariate AR(1)

%WITHIN%

!stability with lag1 (= 1 day)

aa| AFF ON AFF&1;

!hypothesized Path: Parenting predicts affect

ap| AFF ON PAR;

!Variance Affect

AFF;

!Variance Parenting

PAR;

%between%

!Intercepts

[AFF];

[PAR];

[aa] (b1);

[ap] (b2);

!Variance

AFF;

PAR;

aa (a1);

ap (a2);

!correlation between random intercepts and random slopes

aa ap PAR AFF with aa ap PAR AFF;

! Calculate the ratio SD and fixed effect (see Bolger et al)

MODEL CONSTRAINT: NEW (RATIOaa RATIOap);

RATIOaa = sqrt(a1)/b1;

RATIOap = sqrt(a2)/b2; ,

OUTPUT: TECH1 TECH8 TECH4(CLUSTER) standardized (cluster);

PLOT: type = plot1 plot2 plot3;

**TITLE: DSEM SYNTAX FOR MODEL 5**

DATA: FILE = 'DATA.dat'; !read data

VARIABLE:

NAMES =

ID days

PAR AFF; ! Names in Dataset

! ID = ID variable

! days = day in study (1 – 125)

! PAR = Parenting variable (warmth/autonomy support)

! AFF = Positive or Negative Affect

MISSING = ALL (9999); ! Missing Values

USEVAR = PAR AFF; !Variables in this analysis

CLUSTER = ID; ! Variable which identifies participants

LAGGED = AFF(1) PAR(1); ! create lagged variables

TINTERVAL = days(1); !account for unequal spacing due to missing days

ANALYSIS:

TYPE = TWOLEVEL RANDOM;

ESTIMATOR=BAYES;

BITER = (5000)! Min 5000 iterations

PROC=2;

THIN = 2;

MODEL:

! Autoregressive model with time varying covariate AR(1)

%WITHIN%

!stability with lag1 (= 1 day)

aa| AFF ON AFF&1;

pp| PAR ON PAR&1;

!hypothesized Path: Parenting predicts affect

ap| AFF ON PAR&1;

pa| PAR ON AFF&1;

!Variance Affect

AFF;

!Variance Parenting

PAR;

%between%

!Intercepts

[AFF];[PAR];[aa] (b1);[pp];[ap] (b2);[pa];

!Variance

AFF; PAR; aa (a1); pp; ap (a2); aa;

!correlation between random intercepts and random slopes

aa pp ap pa PAR AFF with aa pp ap pa PAR AFF;

! Calculate the ratio SD and fixed effect (see Bolger et al)

MODEL CONSTRAINT: NEW (RATIOaa RATIOap);

RATIOaa = sqrt(a1)/b1;

RATIOap = sqrt(a2)/b2;

OUTPUT: TECH1 TECH8 TECH4(CLUSTER) standardized (cluster);

PLOT: type = plot1 plot2 plot3;

**TITLE: DSEM SYNTAX FOR MODEL 6**

DATA: FILE = 'DATA.dat'; !read data

VARIABLE:

NAMES =

ID days

PAR AFF; ! Names in Dataset

! ID = ID variable

! days = day in study (1 – 125)

! PAR = Parenting variable (warmth/autonomy support)

! AFF = Positive or Negative Affect

MISSING = ALL (9999); ! Missing Values

USEVAR = PAR AFF; !Variables in this analysis

CLUSTER = ID; ! Variable which identifies participants

! LAGGED = AFF(1); ! create lagged variables

! TINTERVAL = days(1); !account for unequal spacing due to missing days

ANALYSIS:

TYPE = TWOLEVEL RANDOM;

ESTIMATOR=BAYES;

BITER = (5000)! Min 5000 iterations

PROC=2;

THIN = 2;

MODEL:

! Autoregressive model with time varying covariate AR(1)

%WITHIN%

!stability with lag1 (= 1 day)

! aa| AFF ON AFF&1;

!hypothesized Path: Parenting predicts affect

ap| AFF ON PAR;

!Variance Affect

AFF;

!Variance Parenting

PAR;

%between%

!Intercepts

[AFF];

[PAR];

![aa] (b1);

[ap] (b2);

!Variance

AFF;

PAR;

!aa (a1);

ap (a2);

!correlation between random intercepts and random slopes

!aa ap PAR AFF with aa ap PAR AFF;

ap PAR AFF with ap PAR AFF:

! Calculate the ratio SD and fixed effect (see Bolger et al)

! MODEL CONSTRAINT: NEW (RATIOaa RATIOap);

MODEL CONSTRAINT: NEW (RATIOap);

!RATIOaa = sqrt(a1)/b1;

RATIOap = sqrt(a2)/b2; ,

OUTPUT: TECH1 TECH8 TECH4(CLUSTER) standardized (cluster);

PLOT: type = plot1 plot2 plot3;

**TITLE: DSEM SYNTAX FOR MODEL WITH MODERATOR**

DATA: FILE = 'DATA.dat'; !read data

VARIABLE:

NAMES =

ID days

PAR AFF MOD; ! Names in Dataset

! ID = ID variable

! days = day in study (1 – 125)

! PAR = Parenting variable (warmth/autonomy support)

! AFF = Positive or Negative Affect

! MOD = Moderator

MISSING = ALL (9999); ! Missing Values

USEVAR = PAR AFF MOD; !Variables in this analysis

CLUSTER = ID; ! Variable which identifies participants

BETWEEN = MOD; ! Moderator does not vary within-person

LAGGED = AFF(1); ! create lagged variables

TINTERVAL = days(1); !account for unequal spacing due to missing days

ANALYSIS:

TYPE = TWOLEVEL RANDOM;

ESTIMATOR=BAYES;

BITER = (5000)! Min 5000 iterations

PROC=2;

THIN = 2;

MODEL:

! Autoregressive model with time varying covariate AR(1)

%WITHIN%

!stability with lag1 (= 1 day)

aa| AFF ON AFF&1;

!hypothesized Path: Parenting predicts affect

ap| AFF ON PAR;

!Variance Affect

AFF;

!Variance Parenting

PAR;

%between%

!Intercepts

[AFF];

[PAR];

[aa] (b1);

[ap] (b2);

!Variance

AFF;

PAR;

aa (a1);

ap (a2);

!correlation between random intercepts and random slopes

aa ap PAR AFF MOD with aa ap PAR AFF MOD;

! Calculate the ratio SD and fixed effect (see Bolger et al)

MODEL CONSTRAINT: NEW (RATIOaa RATIOap);

RATIOaa = sqrt(a1)/b1;

RATIOap = sqrt(a2)/b2; ,

OUTPUT: TECH1 TECH8 TECH4(CLUSTER) standardized (cluster);

PLOT: type = plot1 plot2 plot3;

# Additional Information to Results Section

## Additional Descriptive Statistics

**Table S2**

*Descriptive Statistics and Correlations for Children’s Baseline Measures (N = 159)*

|  | *M* | *SD* | Min - Max | Skewness | Kurtosis | ω | 1 | 2 | 3 | 4 | 5 | 6 |
| --- | --- | --- | --- | --- | --- | --- | --- | --- | --- | --- | --- | --- |
| 1 Neuroticism | 2.71 | 0.74 | 1.00 – 4.83 | 0.16 | -0.42 | .79 |  |  |  |  |  |  |
| 2 Extraversion | 3.43 | 0.70 | 1.00 – 3.83 | -0.59 | 0.58 | .80 | -.35* |  |  |  |  |  |
| 3 Openness | 3.45 | 0.74 | 1.00 – 5.00 | -0.22 | -0.01 | .76 | .16 | .07 |  |  |  |  |
| 4 Agreeableness | 3.78 | 0.53 | 2.33 – 5.00 | -0.09 | -0.29 | .65 | .01 | .13 | .38 |  |  |  |
| 5 Conscientiousness | 3.19 | 0.70 | 1.50 – 5.00 | 0.12 | -0.13 | .80 | -.29*** | .20* | .29*** | .39 |  |  |
| 6 Environmental Sensitivity | 4.59 | 0.74 | 2.67 – 6.75 | 0.20 | 0.05 | .74 | .51*** | -.15 | .38** | .25 | .05 |  |

*Note.* * p <.05, ** p <.01, *** p <.001

## Results of Parent-reported Models

**Table S3**

*Model Results of Dynamic Structure Equation Models (parent-reported need supportive parenting)*

|  | Positive Affect | | |  | Negative Affect | | |
| --- | --- | --- | --- | --- | --- | --- | --- |
|  | Est. | Est. St. | 95% CI |  | Est. | Est. St. | 95% CI |
|  | **Parental Warmth** | | | | | | |
| ***Average within-family*** |  |  |  |  |  |  |  |
| Parental Warmth(t) -> Affect (t) | 0.18 | .12 | **[0.14; 0.22]** |  | -0.14 | -.11 | **[-0.19; -0.10]** |
| Affect (t-1) -> Affect (t) | 0.32 | .32 | **[0.28; 0.36]** |  | 0.26 | .26 | **[0.22; 0.29]** |
|  |  |  |  |  |  |  |  |
| ***Average between-family*** |  |  |  |  |  |  |  |
| Parental Warmth & Affect | 19.22 | .10 | [-11.54; 52.67] |  | -5.15 | -.04 | [-26.26; 15.72] |
|  |  |  |  |  |  |  |  |
| ***Variance within-family*** |  |  |  |  |  |  |  |
| Parental Warmth (t) -> Affect (t) | 0.04 |  | [0.03; 0.06] |  | 0.05 |  | [0.04; 0.07] |
| Affect (t-1) -> Affect (t) | 0.04 |  | [0.03; 0.05] |  | 0.03 |  | [0.02; 0.04] |
|  |  |  |  |  |  |  |  |
| ***Ratio: SD / Fixed Effect (H1)*** |  |  |  |  |  |  |  |
| Parental Warmth (t) -> Affect (t) | **1.11** |  | [0.84; 1.55] |  | **1.57** |  | [1.16; 2.36] |
| Affect (t-1) -> Affect (t) | **0.58** |  | [0.48; 0.72] |  | **0.66** |  | [0.54; 0.84] |
|  | **Autonomy Support** | | | | | | |
| ***Average Within-family*** |  |  |  |  |  |  |  |
| Autonomy Support (t) -> Affect (t) | 0.07 | .07 | **[0.04; 0.10]** |  | -0.06 | -.07 | **[-0.09; -0.04]** |
| Affect (t-1) -> Affect (t) | 0.32 | .32 | **[0.28; 0.36]** |  | 0.26 | .26 | **[0.22; 0.29]** |
|  |  |  |  |  |  |  |  |
| ***Average Between-family*** |  |  |  |  |  |  |  |
| Autonomy Support & Affect | 14.36 | .07 | [-18.54; 48.72] |  | 3.16 | .02 | [-19.35; 26.29] |
|  |  |  |  |  |  |  |  |
| ***Variance within family*** |  |  |  |  |  |  |  |
| Autonomy Support (t) -> Affect (t) | 0.02 |  | [0.01; 0.03] |  | 0.01 |  | [0.01; 0.02] |
| Affect (t-1) -> Affect (t) | 0.04 |  | [0.03; 0.05] |  | 0.03 |  | [0.02; 0.04] |
|  |  |  |  |  |  |  |  |
| ***Ratio: SD / Fixed Effect (H1)*** |  |  |  |  |  |  |  |
| Autonomy Support (t) -> Affect (t) | **1.89** |  | [1.27; 3.43] |  | **1.85** |  | [1.28; 3.13] |
| Affect (t-1) -> Affect (t) | **0.58** |  | [0.48; 0.71] |  | **0.64** |  | [0.52; 0.81] |

*Note*: Est. = unstandardized estimates; Est. St. = standardized estimates for fixed within- and between-family effects, standardized using the STDYX Standardization (Within-Level Standardized Estimates Averaged over Clusters) in M*plus*; Ratio: Random Slope SD / Fixed Effect: a point estimate >0.25 is the criterium we defined as meaningful effect heterogeneity (Bolger et al., 2019); 95% CI = 95% Credibility interval. Bold values indicate significant/meaningful estimates.

## Different Visualization of Variation in Family-specific Effects

**Figure S2**

*Distribution of family-specific estimates*


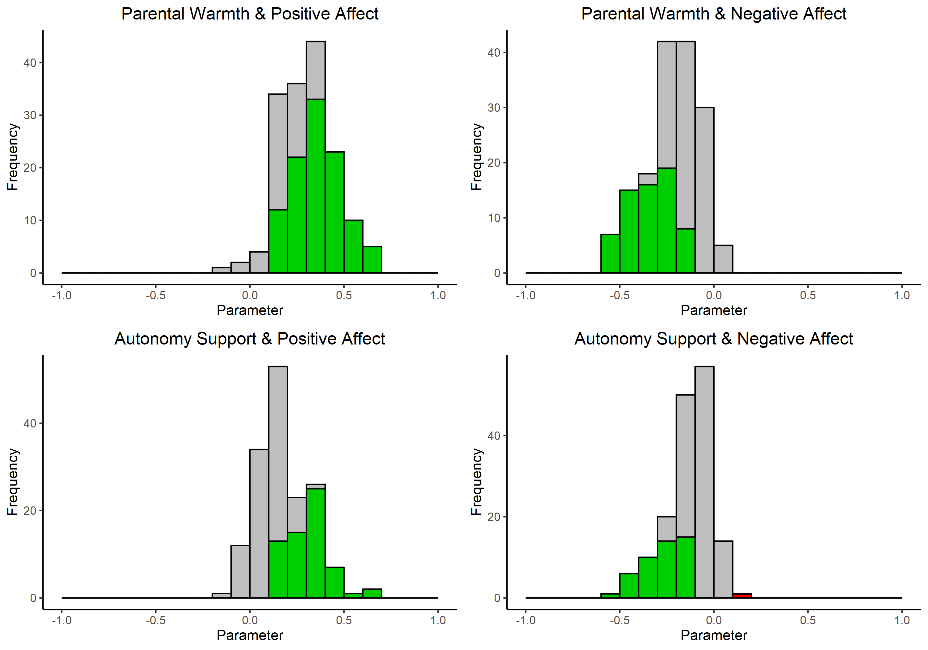


*Note*. green bars indicate participants who are correctly classified, grey bars indicate participants who are ambiguously classified, and red bars identify participants who are incorrectly classified.

**Figure S3**

*Co-Fluctuations of Daily Parental Warmth and Positive Affect in Four Families*


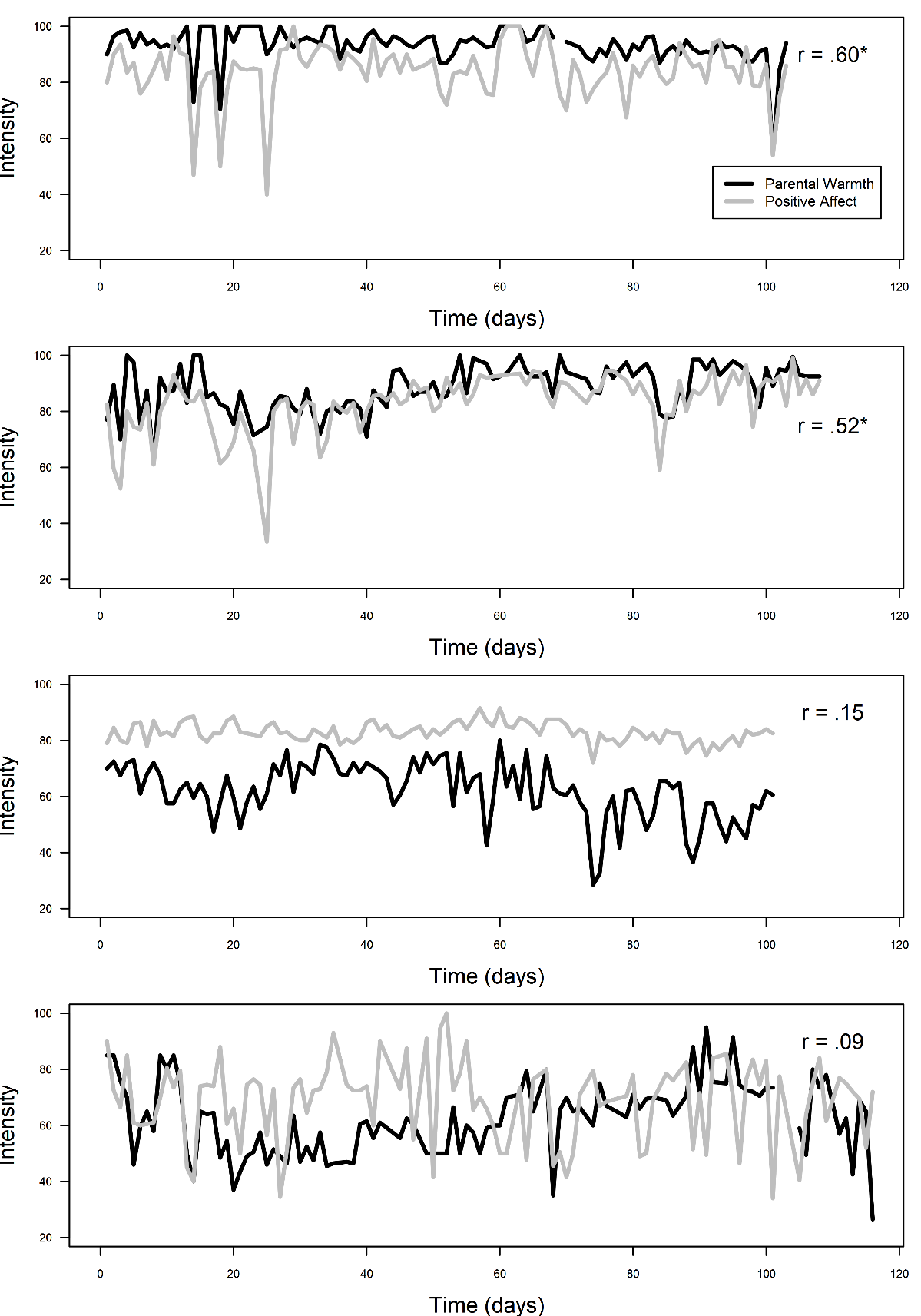


*Note.* * significant family-specific correlation

## Visualization of Association between Family-specific Effects and Environmental Sensitivity

**Figure S4**

*Association between Family-specific Effects and Environmental Sensitivity*


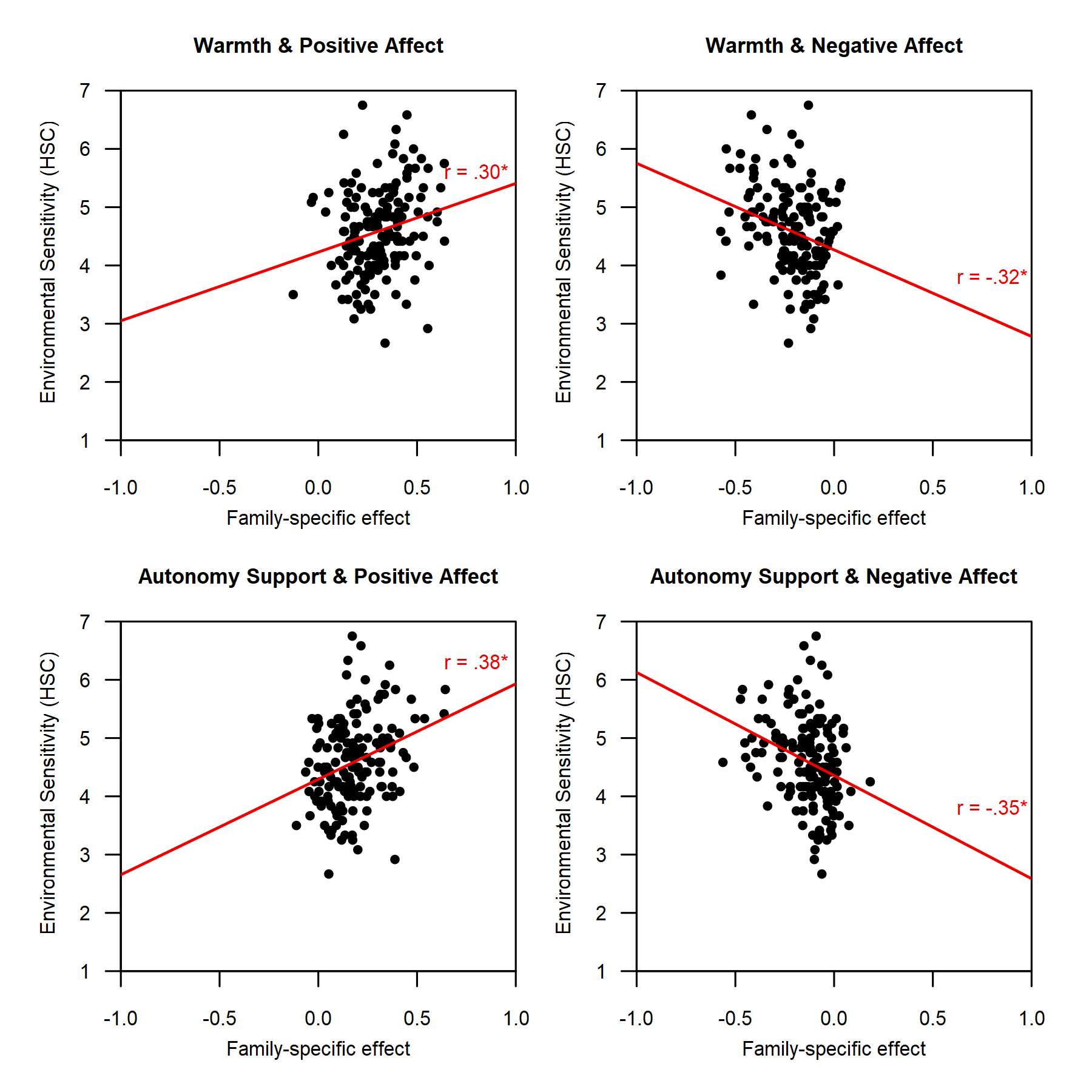


## Sensitivity Analysis

Ten models with slightly different model specifications or data were run as sensitivity analyses to check the robustness of our findings. The results can be found in Table S4 & S5, where models are compared to the main model reported in the article (i.e., Model 1). For each model, the fixed effects, the variance around these effects, the distribution of classification and the extent to which the family specific estimates correlate with the family-specific estimates of Model 1 are compared.

Model results replicate when doubling the number of iterations (Model 2) or excluding participants with less than 50 measurements (*n* = 7; Model 3).

Additionally, models with similar specifications (Model 4: parent-reported parenting, Model 5: lagged affects; Model 6: no control for prior day affect) show similar results. Hypothesis 1 (parenting effects are not uniform but differ in strength) could be replicated in Model 4, Model 5 & Model 6. Hypothesis 2 could be replicated in Model 4, Model 6, and partially in Model 5: Effects of parenting predicting next day’s *positive* *affect* were universal. In model 5 where parenting predicted *negative affect* only 2 – 4 % of family-specific effects were significant, precluding a conclusion of the universality of these effects.

In Model 7 through Model 11, the models were rerun with the same specifications as in Model 1 with a subset of the data: The first 75 days, the first 50 days, the last 50 days, the first 25 days and the first 10 days. The results give an indication if similar results would be achieved in a replication study with a shorter study period. In all models, differences in strength of parenting effects (H1) could be replicated, indicating that with only 10 days of daily diary such results can be obtained. The universality of parenting (H2) could also be confirmed in 18 out of the 20 models. For autonomy support and negative affect with 10 or 25 days only 2% or 5% of participants had significant family-specific effects, which precludes the possibility to decide if H2 should be accepted or rejected. As expected with less power, due to fewer data points, fewer family-specific estimates were significant in models with less data (e.g., warmth and positive affect: 100 days 66% correctly classified, 10 days: 11% correctly classified).

Of special interest is the comparison of Model 8 and Model 9 with each other as well as with Model 1. In Model 8 the first 50 days of the study are analyzed, while in Model 9 the last 50 days are analyzed. On day 51 (15 December 2021) government restrictions were introduced to prevent the spread of COVID-19 in the Netherlands. From Day 1 until Day 50, adults (>= 18 years) should keep a social distance (1.5m) from other people outside their own household and work from home. From Day 51 until Day 125, also children (<18 years) should keep a social distance from other children, schools and sport clubs were closed and public spaces and non-essential shops were closed. Therefore, comparing the first and second half of the study period gives an indication if the lockdown measures might have influenced the results. Results of the first 50 days replicate in the second 50 days. We therefore conclude that the lockdown as well as other factors (e.g., study burden) did not meaningfully affected the process of interest.

**Table S4**

*Sensitivity analysis for Parental Warmth Models*

|  | Model 1 | Model 2 | Model 3 | Model 4 | Model 5 | Model6 | Model 7 | Model 8 | Model 9 | Model 10 | Model 11 |
| --- | --- | --- | --- | --- | --- | --- | --- | --- | --- | --- | --- |
| ***Within-family*** |  |  |  |  |  |  |  |  |  |  |  |
| Warmth(t) -> Pos. Affect (t) | **.30** | **.30** | **.29** | **.12** | **.09**^a^ | **.33** | **.30** | **.28** | **.29** | **.25** | **.23** |
| Pos. Affect (t) -> Pos. Affect (t+1) | **.26** | **.26** | **.26** | **.32** | **.28** | - | **.25** | **.24** | **.21** | **.28** | **.32** |
| ***Between-family*** |  |  |  |  |  |  |  |  |  |  |  |
| Warmth & Pos. Affect | **.58** | **.58** | **.58** | .10 | **.67** | **.51** | **.59** | **.62** | **.60** | **.68** | **.66** |
| ***Ratio: SD / Fixed Effect (H1)*** |  |  |  |  |  |  |  |  |  |  |  |
| Warmth (t) -> Pos. Affect (t) | **0.65** | **0.65** | **0.64** | **1.11** | **1.85**^a^ | **0.62** | **0.67** | **0.82** | **0.76** | **0.82** | **1.06** |
| Pos. Affect (t) -> Pos. Affect (t+1) | **0.66** | **0.66** | **0.66** | **0.58** | **0.63** | - | **0.69** | **0.81** | **0.92** | **0.93** | **0.97** |
| ***Classification (H2)*** |  |  |  |  |  |  |  |  |  |  |  |
| Correctly | 66% | 65% | 66% | 25% | 15% | 69% | 59% | 45% | 40% | 31% | 11% |
| Ambiguously | 34% | 35% | 34% | 75% | 83% | 31% | 41% | 55% | 61% | 69% | 89% |
| Incorrectly | 0% | 0% | 0% | 0% | 2% | 0% | 0% | 0% | 0% | 0% | 0% |
| ***Correlation with Model 1*** |  |  |  |  |  |  |  |  |  |  |  |
| Warmth(t) -> Pos. Affect (t) | **1.00** | **1.00** | **1.00** | **.28** | **.27** | **.95** | **.91** | **.80** | **.71** | **.48** | **.47** |
|  |  |  |  |  |  |  |  |  |  |  |  |
| ***Within-family*** |  |  |  |  |  |  |  |  |  |  |  |
| Warmth(t) -> Neg. Affect (t) | **-.21** | **-.21** | **-.21** | **-.11** | -.03^a^ | **-.22** | **-.21** | **-.20** | **-.22** | **-.19** | **-.21** |
| Neg. Affect (t) -> Neg. Affect (t+1) | **.24** | **.24** | **.24** | **.26** | **.25** | - | **.23** | **.22** | **.20** | **.24** | **.32** |
| ***Between-family*** |  |  |  |  |  |  |  |  |  |  |  |
| Warmth & Neg. Affect | **-.38** | **-.38** | **-.38** | -.04 | **-.38** | **-.37** | **-.36** | **-.35** | **-.42** | **-.34** | **-.31** |
| ***Ratio: SD / Fixed Effect (H1)*** |  |  |  |  |  |  |  |  |  |  |  |
| Warmth (t) -> Neg. Affect (t) | **0.89** | **0.89** | **0.90** | **1.57** | **3.37**^a^ | **0.94** | **0.92** | **1.04** | **0.96** | **1.15** | **1.07** |
| Neg. Affect (t) -> Neg. Affect (t+1) | **0.65** | **0.65** | **0.63** | **0.66** | **0.69** | - | **0.67** | **0.59** | **1.07** | **0.78** | **0.91** |
| ***Classification (H2)*** |  |  |  |  |  |  |  |  |  |  |  |
| Correctly | 41% | 43% | 43% | 17% | 4% | 38% | 36% | 27% | 36% | 18% | 15% |
| Ambiguously | 59% | 57% | 57% | 82% | 96% | 62% | 64% | 73% | 64% | 82% | 85% |
| Incorrectly | 0% | 0% | 0% | 0% | 0% | 0% | 0% | 0% | 0% | 1% | 0% |
| ***Correlation with Model 1*** |  |  |  |  |  |  |  |  |  |  |  |
| Warmth(t) -> Neg. Affect (t) | **1.00** | **1.00** | **1.00** | **.49** | .17 | **.97** | **.92** | **.80** | **.79** | **.51** | **.40** |

*Note.* Model 1 = model in paper, Model 2 = double iterations, Model 3 = participants with 50 or more data points (*N* = 152), Model 4 = parent report, Model 5 = lagged effects (1 day), Model 6 = no control for prior day affect, Model 7 = Data of first 75 days, Model 8 = Data of first 50 days, Model 9 = Data of lockdown (Day 51 til 126), Model 10 = Data of first 25 days, Model 11 = Data of first 10 days

For within- and between-family association the standardized effects are reported. For the variance the standard-deviation-fixed-effects-ratio is reported, for the classification the percent participants per group are given. Bold indicate significant results;

^a^ lagged and not concurrent effects.

**Table S5**

*Sensitivity analysis for Autonomy Support Models*

|  | Model 1 | Model 2 | Model 3 | Model 4 | Model 5 | Model6 | Model 7 | Model 8 | Model 9 | Model 10 | Model 11 |
| --- | --- | --- | --- | --- | --- | --- | --- | --- | --- | --- | --- |
| ***Within-family*** |  |  |  |  |  |  |  |  |  |  |  |
| Autonomy Support (t) -> Pos. Affect (t) | **.18** | **.18** | **.18** | **.07** | **.05**^a^ | **.21** | **.18** | **.19** | **.19** | **.16** | **.16** |
| Pos. Affect (t) -> Pos. Affect (t+1) | **.30** | **.30** | **.30** | **.32** | **.31** | **-** | **.27** | **.25** | **.25** | **.28** | **.35** |
| ***Between-family*** |  |  |  |  |  |  |  |  |  |  |  |
| Autonomy Support & Pos. Affect | **.48** | **.48** | **.49** | .07 | **.48** | **.41** | **.47** | **.46** | **.47** | **.55** | **.51** |
| ***Ratio: SD / Fixed Effect (H1)*** |  |  |  |  |  |  |  |  |  |  |  |
| Autonomy Support (t) -> Pos. Affect (t) | **1.02** | **1.02** | **1.03** | **1.89** | **3.00**^a^ | **0.97** | **1.05** | **1.15** | **1.22** | **1.36** | **1.34** |
| Pos. Affect (t) -> Pos. Affect (t+1) | **0.62** | **0.62** | **0.63** | **0.58** | **0.65** | **-** | **0.65** | **0.76** | **0.87** | **0.94** | **0.92** |
| ***Classification (H2)*** |  |  |  |  |  |  |  |  |  |  |  |
| Correctly | 40% | 40% | 38% | 11% | 12% | 44% | 35% | 28% | 24% | 19% | 10% |
| Ambiguously | 60% | 60% | 62% | 88% | 86% | 55% | 65% | 72% | 75% | 81% | 90% |
| Incorrectly | 0% | 0% | 0% | 1% | 3% | 1% | 0% | 0% | 1% | 0% | 0% |
| ***Correlation with Model 1*** |  |  |  |  |  |  |  |  |  |  |  |
| Autonomy Support (t) -> Pos. Affect (t) | **1.00** | **1.00** | **1.00** | **.27** | **.37** | **.93** | **.93** | **.80** | **.67** | **.64** | **.57** |
|  |  |  |  |  |  |  |  |  |  |  |  |
| ***Within-family*** |  |  |  |  |  |  |  |  |  |  |  |
| Autonomy Support (t) -> Neg. Affect (t) | **-.13** | **-.13** | **-.13** | **-.07** | -.01^a^ | **-.14** | **-0.13** | **-.12** | **-.14** | **-.09** | **-.10** |
| Neg. Affect (t) -> Neg. Affect (t+1) | **.25** | **.25** | **.25** | **.26** | **.25** | **-** | **0.24** | **.22** | **.20** | **.23** | **.32** |
| ***Between-family*** |  |  |  |  |  |  |  |  |  |  |  |
| Autonomy Support & Neg. Affect | **-.30** | **-.30** | **-.30** | .02 | **-0.30** | **-.29** | **-0.25** | **-.26** | **-.35** | **-.20** | **-0.26** |
| ***Ratio: SD / Fixed Effect (H1)*** |  |  |  |  |  |  |  |  |  |  |  |
| Autonomy Support (t) -> Neg. Affect (t) | **1.28** | **1.25** | **1.30** | **1.85** | **7.82**^a^ | **1.29** | **1.33** | **1.50** | **1.75** | **1.37** | **1.42** |
| Neg. Affect (t) -> Neg. Affect (t+1) | **0.65** | **0.66** | **0.65** | **0.64** | **0.69** | **-** | **0.70** | **0.58** | **0.96** | **0.74** | **0.88** |
| ***Classification (H2)*** |  |  |  |  |  |  |  |  |  |  |  |
| Correctly | 29% | 27% | 29% | 13% | 2% | 31% | 25% | 15% | 23% | 5% | 2% |
| Ambiguously | 70% | 72% | 70% | 87% | 98% | 68% | 75% | 85% | 76% | 95% | 98% |
| Incorrectly | 1% | 1% | 1% | 0% | 0% | 1% | 0% | 0% | 1% | 0% | 0% |
| ***Correlation with Model 1*** |  |  |  |  |  |  |  |  |  |  |  |
| Autonomy Support (t) -> Neg. Affect (t) | **1.00** | **1.00** | **1.00** | **.36** | **.21** | **.97** | **.92** | **.78** | **.75** | **.57** | **.40** |

*Note.* Model 1 = model in paper, Model 2 = double iterations, Model 3 = participants with 50 or more data points (*N* = 152), Model 4 = parent report, Model 5 = lagged effects (1 day), Model 6 = no control for prior day affect, Model 7 = Data of first 75 days, Model 8 = Data of first 50 days, Model 9 = Data of lockdown (Day 51 til 126), Model 10 = Data of first 25 days, Model 11 = Data of first 10 days

For within- and between-family association the standardized effects are reported. For the variance the standard-deviation-fixed-effects-ratio is reported, for the classification the percent participants per group are given. Bold indicate significant results;

^a^ lagged and not concurrent effects

**Figure S5**

*Distribution of family-specific estimates of parent-reported models (Model 4)*


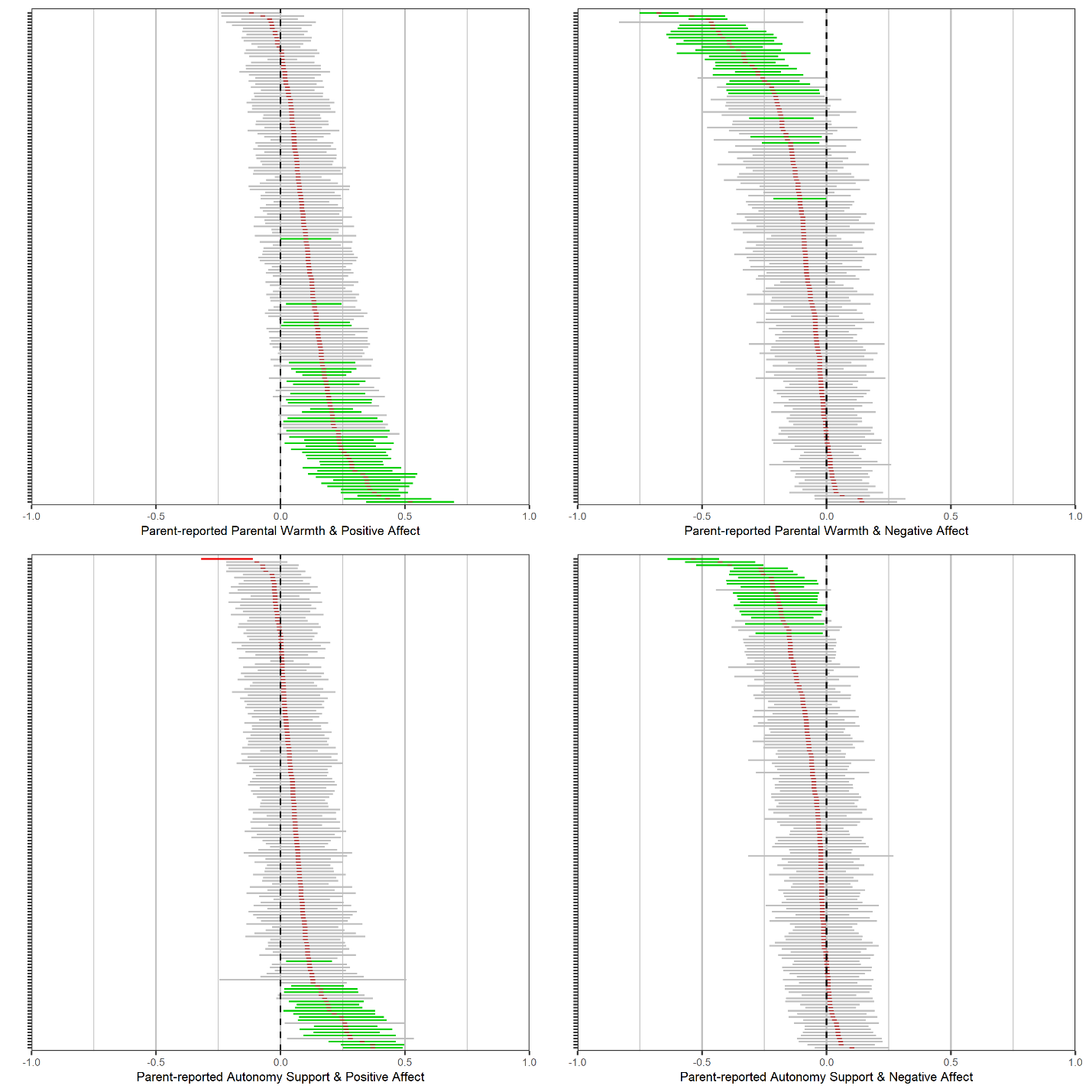


**Figure S6**

*Distribution of family-specific estimates of lagged-effects (Model 5)*


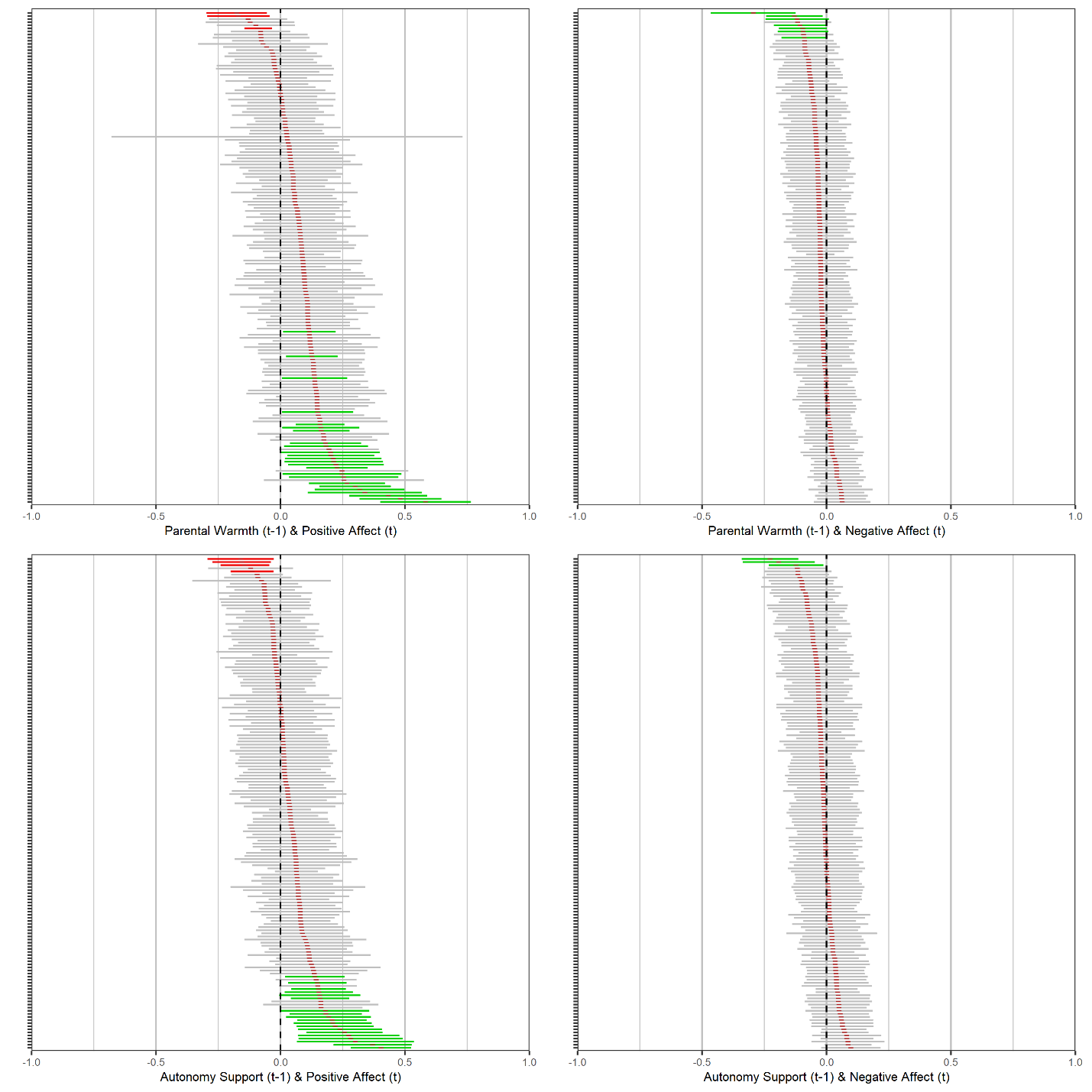


## Exploration of Incorrectly Classified Participant

In the model analyzing the association between autonomy support and negative affect, one participant was “incorrectly classified”. This participant had a significant positive association (*r* = .18), even though we theoretically expected that autonomy support and negative affect should be negatively associated. Data of this participant (*t* = 82) were visually inspected (see Figure S7, upper panel). Sensitivity analysis confirmed that the positive correlation was driven by two outliers (Day 60 & 99), where the participant had a value of 100 (max. of scale) for both autonomy support and negative affect. Removing these two days resulted in a non-significant correlation of *r* = -.04 (see Figure S7 lower panel and Figure S8). As the participant also indicated on these days extreme high values on other scales (Day 60: warmth & positive affect, Day 99: warmth), and did not report on any special events on these days, we suspect this participant carelessly responded on at least these two days. Therefore, we question if the significant unexpected correlation reflects this participants’ true value.

**Figure S7**

*Data of incorrectly classified participant*


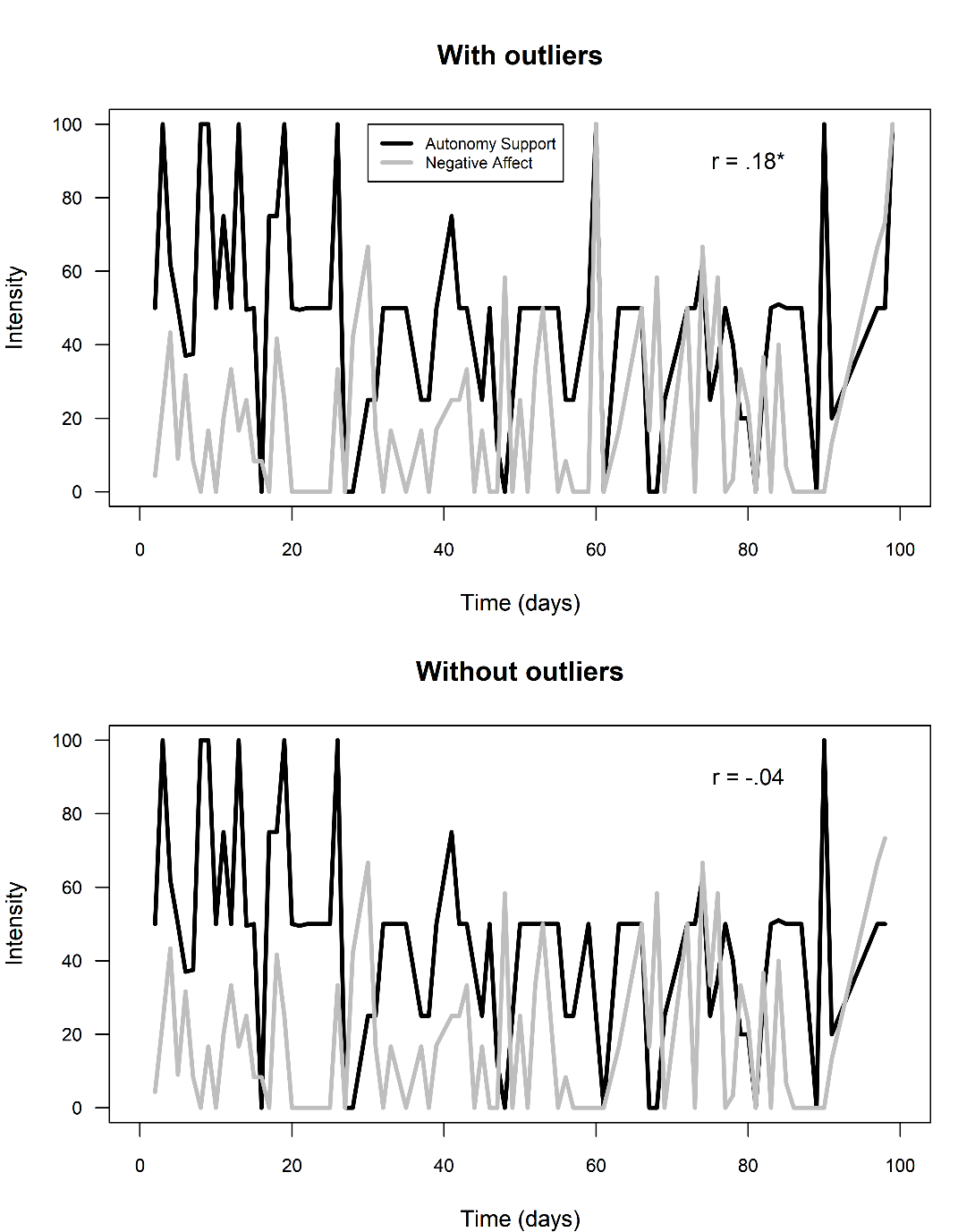


*Note*. Upper panel depicts all data (with outliers), lower panel depicts data without two days, which were identified as outliers.

**Figure S8**

*Scatterplot of incorrectly classified participant*


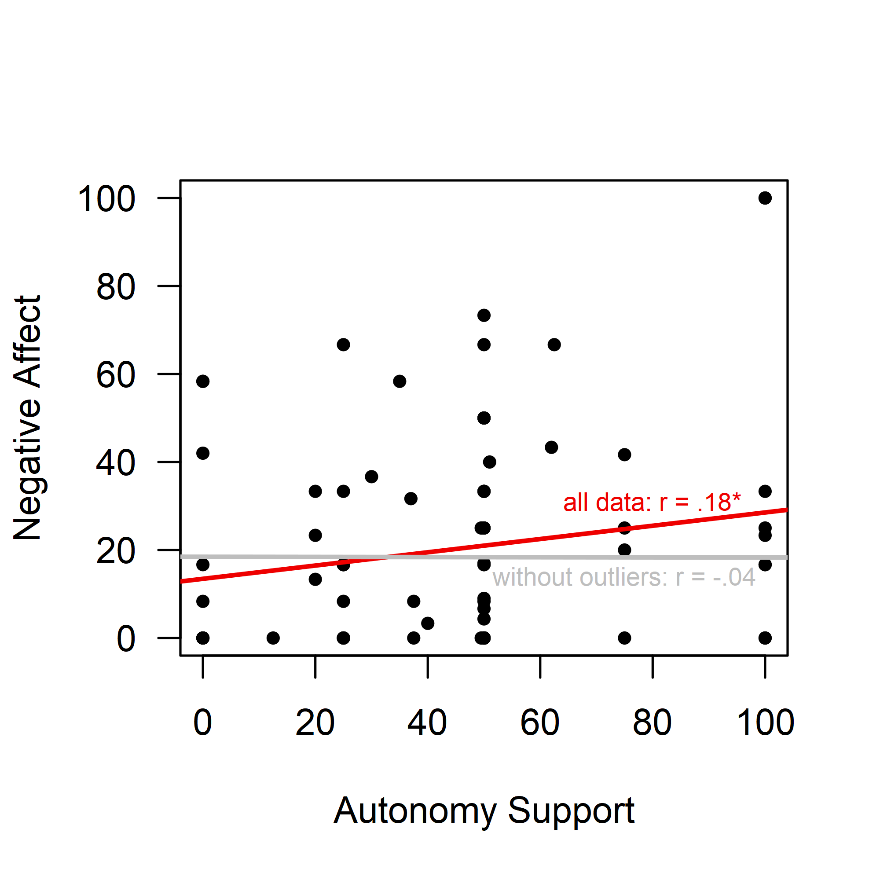


## Further Exploratory Analysis

The analysis revealed that there were differences in the effect size of parenting on child affect between families. For some families the effects were stronger than for others. We explored if these differences predicted divergent developmental patterns. In the study before during and after the 100 days, depressive symptoms and well-being were assessed 5 times (every 3 month for a year). Linear change in depressive symptoms and well-being was associated with the differences in family-specific effects. The constructs were assessed as follows:

**Well-being**. Children answered in all five longitudinal questionnaires Stirling’s Children’s Well-being Scale (Liddle & Carter, 2015), which consists of 6 items (e.g., “I enjoy what each new day brings.”). Items are rated on a 5-point Likert scale (1 “*Never*” to 5 “*All the time*”). The scale showed good internal consistency (ω between: .82 to .88).

**Depressive symptoms**. Children answered in all five questionnaires the short version of Reynolds Adolescent Depression Scale (RADS-2), which consists of 10 items (e.g., “I’ve felt like a bad person”). Items were answered on a 4-point Likert scale (1 “*Almost never*” to 4 “*Often*”). The scale showed an excellent internal consistency (ω between: .89 to .92)

In Table S6 the descriptive statistics of these measures are displayed.

**Table S6**

*Descriptive Statistics for Longitudinal measures*

|  | *N* | *M* | *SD* | Min - Max | Skew | Kurtosis | ω |
| --- | --- | --- | --- | --- | --- | --- | --- |
| Depressive Symptoms (Q1) | 157 | 1.78 | 0.64 | 1.00 – 3.50 | 0.90 | -0.08 | .90 |
| Depressive Symptoms (Q2) | 147 | 1.96 | 0.64 | 1.00 – 3.80 | 0.58 | -0.42 | .90 |
| Depressive Symptoms (Q3) | 118 | 1.98 | 0.68 | 1.00 – 3.80 | 0.74 | -0.28 | .91 |
| Depressive Symptoms (Q4) | 108 | 1.91 | 0.65 | 1.00 – 3.70 | 0.64 | -0.50 | .89 |
| Depressive Symptoms (Q5) | 119 | 1.85 | 0.70 | 1.00 – 3.90 | 0.87 | 0.04 | .92 |
| Well-being (Q1) | 157 | 3.69 | 0.53 | 2.17 – 5.00 | -0.46 | 0.29 | .82 |
| Well-being (Q2) | 146 | 3.56 | 0.62 | 1.50 – 5.00 | -0.68 | 1.00 | .88 |
| Well-being (Q3) | 117 | 3.49 | 0.66 | 1.00 – 4.67 | -1.01 | 1.78 | .88 |
| Well-being (Q4) | 107 | 3.57 | 0.58 | 2.17 – 4.83 | -0.18 | -0.33 | .87 |
| Well-being (Q5) | 116 | 3.60 | 0.60 | 2.00 – 4.83 | -0.61 | 0.20 | .87 |

Using the syntax below, the links between strength of family-specific effects were correlated with the long-term trajectories of well-being and depressive symptoms. No significant associations were found (see Table S7).

**Table S7.**

*Correlates of between-person differences in within-person parenting effects*

|  | PW & PA | PW & NA | AS & PA | AS & NA |
| --- | --- | --- | --- | --- |
| Changes in well-being | .12 | .15 | .04 | -.06 |
| Changes in depression | .14 | -.18 | .19 | -.11 |

*Note.* PW = Parental Warmth, PA = Positive Affect, NA = Negative Affect, AS = Autonomy Support.

**TITLE: DSEM SYNTAX FOR MODEL WITH CHANGE OVER TIME**

DATA: FILE = 'DATA.dat'; !read data

! Syntax adapted from Brose et al 2021 (<https://osf.io/b8udv/>)

VARIABLE:

NAMES =

ID days

PAR AFF WB1 WB2 WB3 WB4 WB5; ! Names in Dataset

! ID = ID variable

! days = day in study (1 – 125)

! PAR = Parenting variable (warmth/autonomy support)

! AFF = Positive or Negative Affect

! WB1 – WB5 = Wellbeing/Depression assessed every 3 month

MISSING = ALL (9999); ! Missing Values

USEVAR = PAR AFF WB1 WB2 WB3 WB4 WB5; !Variables in this analysis

BETWEEN = WB1 WB2 WB3 WB4 WB5;

CLUSTER = ID; ! Variable which identifies participants

LAGGED = AFF(1); ! create lagged variables

TINTERVAL = days(1); !account for unequal spacing due to missing days

ANALYSIS:

TYPE = TWOLEVEL RANDOM;

ESTIMATOR=BAYES;

BITER = (5000)! Min 5000 iterations

PROC=2;

THIN = 2;

MODEL:

! Autoregressive model with time varying covariate AR(1)

%WITHIN%

!stability with lag1 (= 1 day)

aa| AFF ON AFF&1;

!hypothesized Path: Parenting predicts affect

ap| AFF ON PAR;

!Variance Affect

AFF;

!Variance Parenting

PAR;

%between%

!Intercepts

[AFF];

[PAR];

[aa] (b1);

[ap] (b2);

!Variance

AFF;

PAR;

aa (a1);

ap (a2);

! modeling of linear growth of wellbeing

! individuals are modeled to differ in l=level and s=slope of wellbeing/depression

l s | WB1@0 WB2@1 WB3@2 WB4@3 WB5@4;

!Intercepts

[l];

[s];

!Variance

l;

s;

l with s;

!integration of state dynamics and trait change:

!s WITH ap;

!correlation between random intercepts and random slopes

aa ap PAR AFF l s with aa ap PAR AFF l s;

! Calculate the ratio SD and fixed effect (see Bolger et al)

MODEL CONSTRAINT: NEW (RATIOaa RATIOap);

RATIOaa = sqrt(a1)/b1;

RATIOap = sqrt(a2)/b2; ,

OUTPUT: TECH1 TECH8 TECH4(CLUSTER) standardized (cluster);

PLOT: type = plot1 plot2 plot3;
